# Ancient DNA from mastics solidifies connection between material culture and genetics of mesolithic hunter–gatherers in Scandinavia

**DOI:** 10.1038/s42003-019-0399-1

**Published:** 2019-05-15

**Authors:** Natalija Kashuba, Emrah Kırdök, Hege Damlien, Mikael A. Manninen, Bengt Nordqvist, Per Persson, Anders Götherström

**Affiliations:** 1 Museum of Cultural History, University of Oslo, P.O. Box 6762. St. Olavs Plass, NO-0130 Oslo, Norway; 2 Department of Archaeology and Ancient History, Uppsala University, P.O. Box 626, SE-751 26 Uppsala, Sweden; 3 Archaeological Research Laboratory, Department of Archaeology and Classical Studies, Stockholm University, SE-106 91 Stockholm, Sweden; 4 Foundation War-Booty Site Finnestorp, Klarinettvägen 75, SE-434 75 Kungsbacka, Sweden

**Keywords:** Genomics, Population genetics

## Abstract

Human demography research in grounded on the information derived from ancient DNA and archaeology. For example, the study on the early postglacial dual-route colonisation of the Scandinavian Peninsula is largely based on associating genomic data with the early dispersal of lithic technology from the East European Plain. However, a clear connection between material culture and genetics has been lacking. Here, we demonstrate that direct connection by analysing human DNA from chewed birch bark pitch mastics. These samples were discovered at Huseby Klev in western Sweden, a Mesolithic site with eastern lithic technology. We generated genome-wide data for three individuals, and show their affinity to the Scandinavian hunter–gatherers. Our samples date to 9880-9540 calBP, expanding the temporal range and distribution of the early Scandinavian genetic group. We propose that DNA from ancient mastics can be used to study environment and ecology of prehistoric populations.

## Abstract (web summary)

Natalija Kashuba et al. analysed human DNA from ancient chewing gums collected from western Sweden. They provide genomic information for three Scandinavian hunter-gatherers, expanding the range of this genetic group and linking them to eastern lithic technologies.

## Introduction

Writing human history using lines of evidence from both ancient DNA and archaeology has been debated since the first DNA-sequence was recovered from human remains^1–3^. Central to this discussion is that ancient human skeletal material often comes from ritual contexts, many times without associated artefacts and therefore lacks any clear connection to the material that archaeologists most often use to study the life of past societies. Earlier aDNA studies suggest the presence of three genetic groups in early postglacial Europe: Western hunter–gatherers (WHG), Eastern hunter–gatherers (EHG), and Scandinavian hunter–gatherers (SHG)^4^. The SHG have been modelled as a mixture of WHG and EHG^4–6^. SHG is genetically the most diverse, suggested to be a consequence of an immigration which took place around 10,300 calBP^6^. This is consistent with the rich archaeological evidence of a dual-route human dispersal into the Scandinavian Peninsula at the end of the latest Ice Age: first migration from the south, which took place at c. 11,500 calBP, and a second one from the northeast, detected at about 10,300 calBP^7–10^. These migrations are associated with differing lithic technological traditions, the one from the latter migration being connected to the pressure blade technology, known in preceding centuries from the East European Plain, Karelia, and Finland, and suggested to have rapidly spread into Scandinavia along a north-eastern route. The connection between a migration from the north-east into Scandinavia and the technological change in the form of pressure blade technology, remains a suggestion, as none of the SHG individuals studied for aDNA have been directly linked to the early eastern blade production technology.

Here, we suggest that a solution to this problem lies in obtaining human aDNA directly from the remnants of material culture, more specifically from chewing gum, masticated lumps, often with imprints of teeth and/or fingers^11^. The material we use is made of birch bark pitch, which is known to have been used as an adhesive substance in lithic tool technology from the Middle Palaeolithic onward in many parts of Eurasia^12–16^, but also for recreational purposes^17,18^ and as a cement for mending and sealing wood and ceramic vessels^19,20^. A variety of substances with similar properties, such as resins from a variety of coniferous trees^21–23^, natural bitumen^24,25^, as well as spinifex, chicle, and other plant gums^26–28^, are also known to have been used in analogous ways in many parts of the world, but their ability to preserve ancient DNA remains to be tested. In this study, we investigate the relationship between Stone Age populations and cultural traits expressed in lithic technology in Scandinavia.

We explore the connection between the demography and material culture of Scandinavian hunter–gatherers by studying mastics and remains of lithic tool production from the Mesolithic Huseby Klev site. At this location in western Sweden, chewing gums and other pieces of mastic, together with lithic remains, are found in a temporally well-defined context, the deep pit excavation trench, dated to c. 10,040–9610 calBP. Analysis of the lithic material shows that the eastern tool technology was used already at an early phase of the site. We consequently generated genome-wide data from mastics representing three individuals, and use the results to reevaluate the earlier suggested co-dispersal of humans and eastern lithic technology.

## Results

### Extracting and authentication of aDNA from ancient mastics

**Fig. 1:**
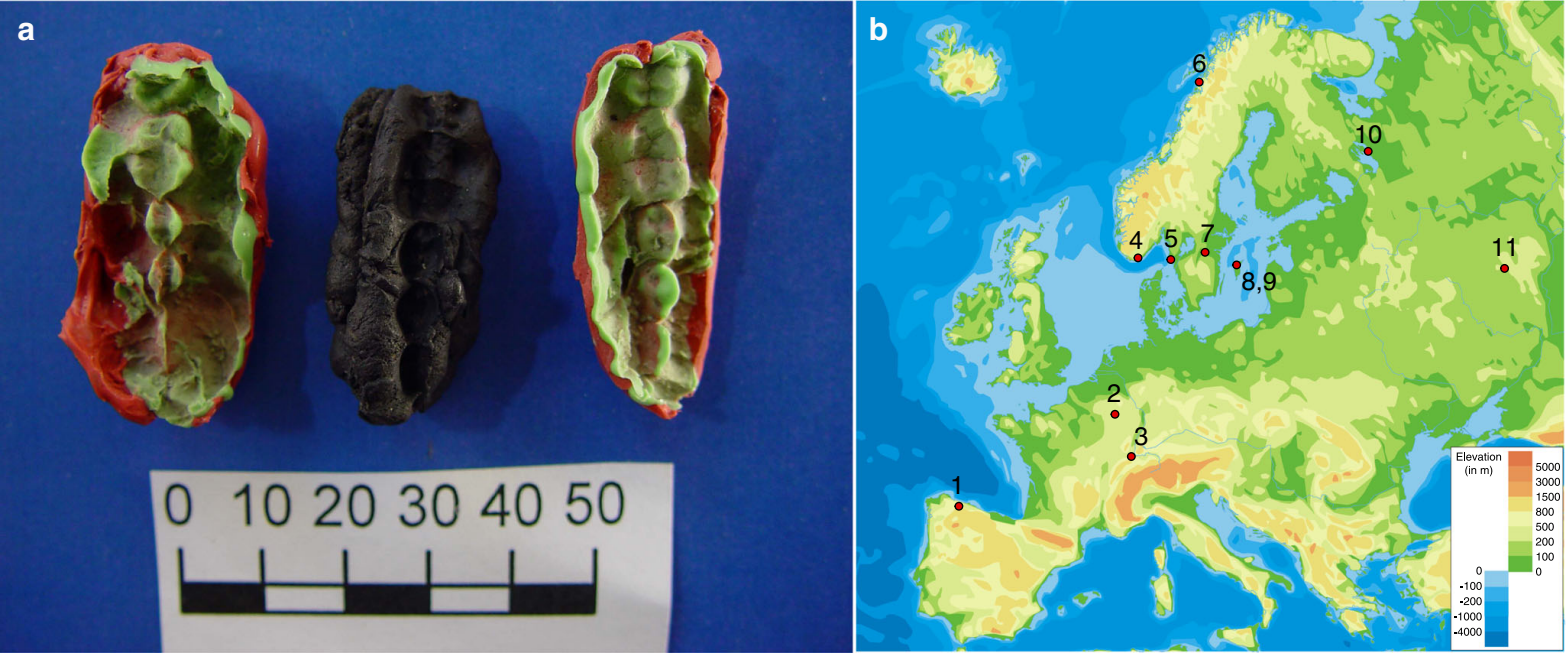
The studied material and its origin. **a** One of the chewing gums from Huseby Klev, (Fynd 2037), with two plastelina casts for each side. The cast to the left captures several teeth imprints from the left side of the maxilla, the one to the right is of the corresponding teeth from the mandible. The presence of the second molar and analysis of tooth wear suggest that the individual, who left these imprints was in the early teens (12–14 years old)^11^. Scale bar: 50 mm (photo by Verner Alexandersen). **b** The location of the sites, genomes from which were used in this study. 1—LaBrana; 2—Loschbour; 3—Bichon; 4—Hummervikholmen; 5—Huseby Klev; 6—Steigen; 7—Motala; 8—Stora Bjers; 9—Stora Förvar; 10—Yuzhnyy Oleni Ostrov; 11—Samara

We performed DNA extraction on eight mastic samples from Huseby Klev (Fig. 1, Supplementary Note 1, Supplementary Data 3). First, we used Yang-Urea extraction following the modifications employed by Svensson et al.^29^ of the published protocol^30^. We named the successful samples ble004, ble007, and ble008. For the ble008 sample we tested one more extraction method using an extraction kit designed to process samples with high-inhibitor content (QIAamp PowerFecal DNA Kit, Qiagen), with slight modification to the protocol provided with the kit (described in the Methods chapter). Blunt-end repaired libraries were built on the concentrated DNA^31^, four of which were UDG treated (for ble004 sample). The libraries were amplified using polymerase chain reaction (PCR) and shotgun sequenced at the Science for Life laboratories (SciLife) in Stockholm, on Illumina Hiseq X platform. Initial tests showed that the individual libraries from Yang-Urea extracts contained authentic ancient human DNA, ranging from 2 to 8% (Supplementary Table 1). The library built on the PowerFecal DNA Kit extract contained 23% endogenous DNA. The tenfold increase in endogenous DNA content makes the PowerFecal DNA extraction kit a valid approach to process mastic samples. By repeating the process with successful extracts, we obtained genome-wide data from three of the mastic pieces, ranging from 0.1× to 0.49× coverage (Table 1). We merged individual libraries using *SAMtools*^32^ and calculated library statistics (before and after PMD filtering, Supplementary Tables 2 and 3) and produced damage plots (Supplementary Fig. 1).

**Table 1:** The library properties for *ble* samples after processing and pmd filtering

Sample	ble004	ble007	ble008
Human sequences	6,463,893	5,802,458	18,393,636
Read length (mean)	66.95	76.98	95.57
Nuclear genome coverage	0.11	0.10	0.49
MT contamination estimate	3.08	1.23	8.61
X chromosome contamination estimate	–	−0.041421×	–
MT haplogroup	U5a2d	U5a2d	U5a2d
Xseq	261,823	170,651	866,414
Yseq	22,093	69,025	26,051
Sex	XX	XY	XX
Number of SNP (compared with Human Origins dataset)	20,103	19,665	52,211

Statistic for ble004 is for merged damage repair and blunt-end repair libraries, ble007 is for blunt-end-repair merged libraries, ble008 is for merged libraries made from extracts produced with different protocols (Supplementary Table 1, Methods)

### Contamination and pmd filtering

We estimated mitochondrial contamination rates using near-private consensus alleles as described by Green et al.^33^ To exclude the effects of sequencing errors, we used bases that have a base-call and mapping quality score of more than Q30. Also, we filtered the positions where we detected transition patterns to compensate the post-mortem damage (Supplementary Table 4). We used the *PMDtools* software to filter out the possible contaminant sequences^34^. This tool compares each aDNA sequence to its modern counterpart to calculate deamination specific nucleotide transitions and assigns a *pmd score*. This score is used to evaluate the authenticity of the sequence. We set the *pmd* threshold to 0 and removed contaminant DNA sequences below this threshold (Supplementary Figs. 2–6). After removing the potential contaminants from the merged libraries, we re-calculated the library statistics, deamination patterns, and MT contamination estimates to analyse the authenticity of the dataset. Deamination patterns (Supplementary Fig. 1) and MT contamination rates (Table 1, Supplementary Tables 4 and 5) present a strong case that the aligned data is authentic and represents the individuals that chewed the ancient mastic (Supplementary Tables 3 and 5).

### Relationships between ancient individuals

We used READ^35^, to explore kinship between the individuals. READ compares the non-overlapping 1 Mb segments in the genome and calculates the nonidentical allele ratio between the samples (P0). Lower P0 values mean more shared chromosomal segments. We confirmed that none of the genomes are identical to each other (Supplementary Table 6). We also found that ble004 and ble007 have a possible second degree relationship. However, it should be noted that using three individuals for analysis is not recommended for this tool, and we refrain from using this result in further discussion. In summary, we can confirm that we sequenced DNA from three distinct individuals.

### Mitochondrial DNA

We used *samtools mpileup* command to create mitochondrial consensus sequences using the nucleotides that have a base-call and mapping quality score of more than Q30. We assigned mitochondrial haplogroups with HaploFind^36^ and HaploGrep 2^37^ (Table 1). We reviewed the results with PhyloTree (build 17)^38^. Mitochondrial genomes from all three individuals belong to the U5a2d haplogroup. ble008 was assigned to U5a2 by HaploGrep 2, but the same sequence is assigned to U5a2d haplogroup by HaploFind and we accept this result (Supplementary Excel Table 1). The mitochondrial U5a2d haplogroup is consistent with earlier published results for ancient individuals from Scandinavia, U5a being the most common within SHG. Of the 16 Mesolithic individuals from Scandinavia published prior to our study, seven belong to the U5a haplogroup, and nine share the U2 and U4 haplogroups^5,6^ (Table 1). The library properties for *ble* samples after processing and pmd filtering. Statistic for ble004 is for merged damage repair and blunt-end repair libraries, ble007 is for blunt-end-repair merged libraries, ble008 is for merged libraries made from extracts produced with different protocols (Supplementary Table 1, Methods)

### Demographic history and population genomics

**Fig. 2:**
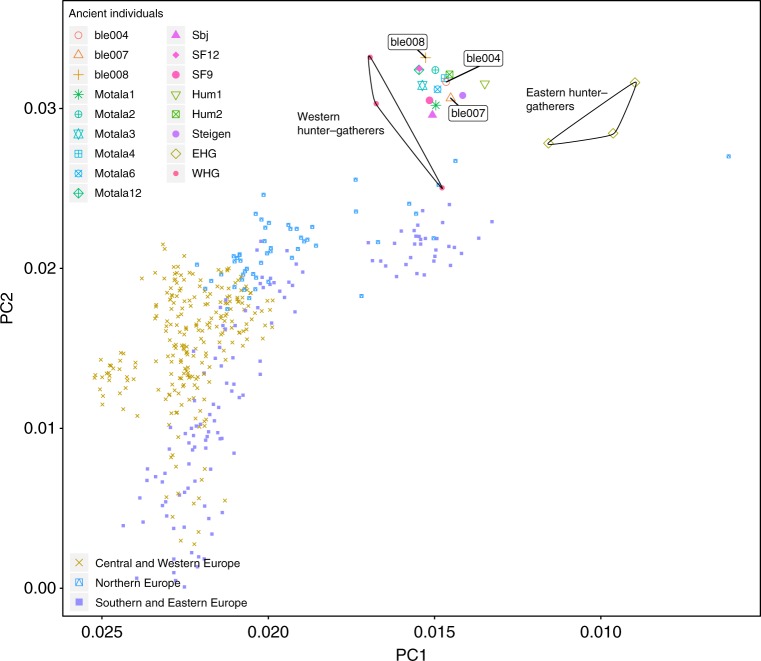
Principal component analysis of the Huseby Klev individuals within the diversity of Mesolithic individuals from Europe. The magnified section incaptures BLE individuals’ relation to Western hunter–gatherer (WHG), Eastern hunter–gatherer (EHG) and Scandinavian hunter–gatherer (SHG) individuals (Supplementary Table 7)

**Fig. 3:**
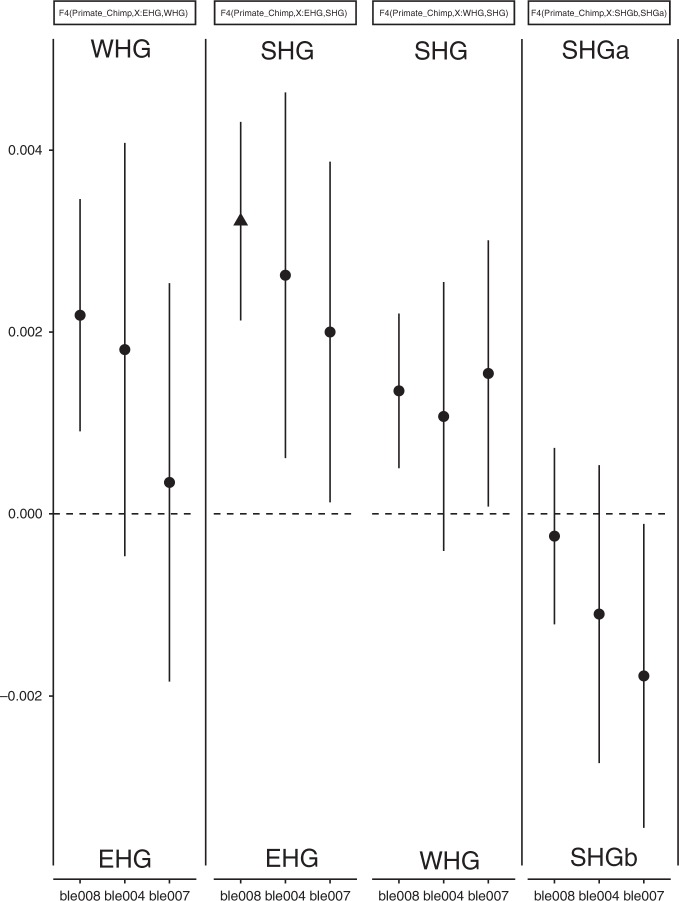
Results of relative allele sharing (F4) test between Huseby Klev individuals and ancient population groups (the triangle marks the significant result in deviation from zero). WHG, Western hunter–gatherers; EHG, Eastern hunter–gatherers; SHG, Scandinavian hunter–gatherers

To explore the demographic history of the ancient individuals, we curated a set of ancient genomes (Supplementary Table 7) and compared with the publicly available Human Origins SNP reference set^39,40^. This set contains 594,924 single nucleotide polymorphisms (SNPs) from 2404 modern individuals and 203 different worldwide populations. We coded deamination patterns as missing data to compensate for possible biases caused by deamination patterns. We used principal component analysis to acquire an overview of the affinity of the ble individuals with selected ancient and modern populations^41^(Fig. 2). We merged ancient individuals with the Human Origins reference set, coded nucleotide transitions as missing data, and used Procrustes transformation to project ancient individuals on the principal component space (Supplementary Fig. 7, Supplementary Table 7). The ancient individuals are divided into three groups. The WHG, include individuals from the sites of LaBrana, Loschbour and Bichon; the EHG, include Yuzhnyy Oleni Ostrov and Samara; and the SHG, include Norwegian Steigen and Hummervikholmen and Swedish Motala, Stora Bjers and Stora Förvar. The projected ancient individuals (WHG, EHG and SHG cluster) show close affinity to modern day North, East and Western European populations, and *BLE* individuals from Huseby Klev (the earliest among the SHG) cluster with the ancient genomes originating from Scandinavia^4^. Our three samples are located between the two Hummervikholmen individuals (Norway), and the Stora Förvar SF9/12 (Sweden), all three with dates earlier than c. 9000 calBP. By reproducing EHG and WHG populations in this plot^6^, we confirm the close affinity of ancient individuals from Scandinavia to WHG and EHG (Fig. 2). To evaluate the relationship between the Huseby Klev individuals and other Mesolithic Scandinavians, we examined the relative shared allele frequencies and estimated shared drift among populations via performing F3 and F4 tests. These tests propose formal statistical frameworks to study the patterns of allele frequency correlation across populations^40^. We first tested the relative allele sharing of BLE individuals between EHG and WHG groups. Results show a high contribution of WHG ancestry to BLE individuals (the highest contribution observed in ble008 individual F4: 0.022, *Z*-score 1.8, Fig. 3). We compared the contribution between ancestry of EHG to SHG and WHG to SHG for BLE individuals. Results show that all tested individuals have relative high-allele sharing with the SHG group, with ble008 individual showing the significant value (F4: 0.03, *Z*-score: 2.95). We divided the SHG group into two groups: SHGa and SHGb, ancient individuals found in contemporary Norway (Steigen and Hummervikholmen) and Sweden (Motala, Stora Bjers and Stora Förvar), respectively. We based this on both the geographical distribution and the previous studies demonstrating the close relation of SHGa to EHG group and SHGb to WHG group^6^. It should be noted that the *Z*-score values for our samples are low for ble004 and ble007, due to low amount of SNPs for these individuals. Moreover, the F3 tests also support the affinity of BLE individuals to SHG group. To further explore the demography within the SHG group, we compared the ancestry of BLE individuals within SHGa and SHGb groups. Both F3 and F4 tests suggest a trend to a high relative shared drift between BLE individuals and the SHGb group (Supplementary Excel Table 2, for F4 and Supplementary Excel Table 3 for F3 tests); however, a symmetrical relationship between BLE and both SHGb and SHGa cannot be ruled out, since only one of the individuals has shown considerable deviation towards any of the groups.

**Fig. 4:**
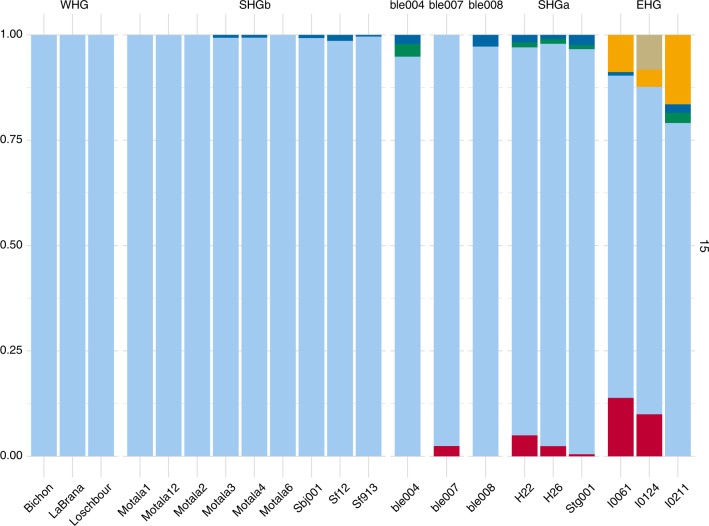
Admixture analysis showing the major mode for *K* = 15. The figure represents 11 runs out of 20 replicates (Greedy algorithm ran with the Jaccard distance and a 0.97 similarity threshold). WHG, Western hunter–gatherers; EHG, Eastern hunter–gatherers; SHG, Scandinavian hunter–gatherers

To further explore our findings from the D and F4 tests, we used the model-based clustering algorithm admixture^42^, which is helpful when exploring the genetic components in a given dataset. The essence of this algorithm is to calculate the admixture proportions as a parameter of a model. The results show that starting from K15, while there is a similarity in genetic elements between the BLE individuals and SHGa, we do not see the red and blue/green component appearing at the same time. Since our best sequenced individual is ble008, which lacks both red and green component, we interpret that the BLE individuals and SHGb population have more genetic similarity (Fig. 4, Supplementary Fig. PDF 1, Fig. PDF 2).

### Lithics results

The technological analysis conducted for the purpose of this study, shows that the lithic artefacts from the deep pit display clear affiliation with the eastern pressure blade technology, as documented from a large number of sites in northern and western Scandinavia, eastern Fennoscandia and the East European Plain^8–10,43^. No artefacts diagnostic to the preceding Early Mesolithic blade technology, that would indicate chronological or technological mixing, were observed (Supplementary Note 1). Based on the composition of lithic artefact types, the site appears to represent a production site to which lithic raw materials, in their more or less unworked condition, were transported, and where initial core preparation and exploitation was performed.

**Fig. 5:**
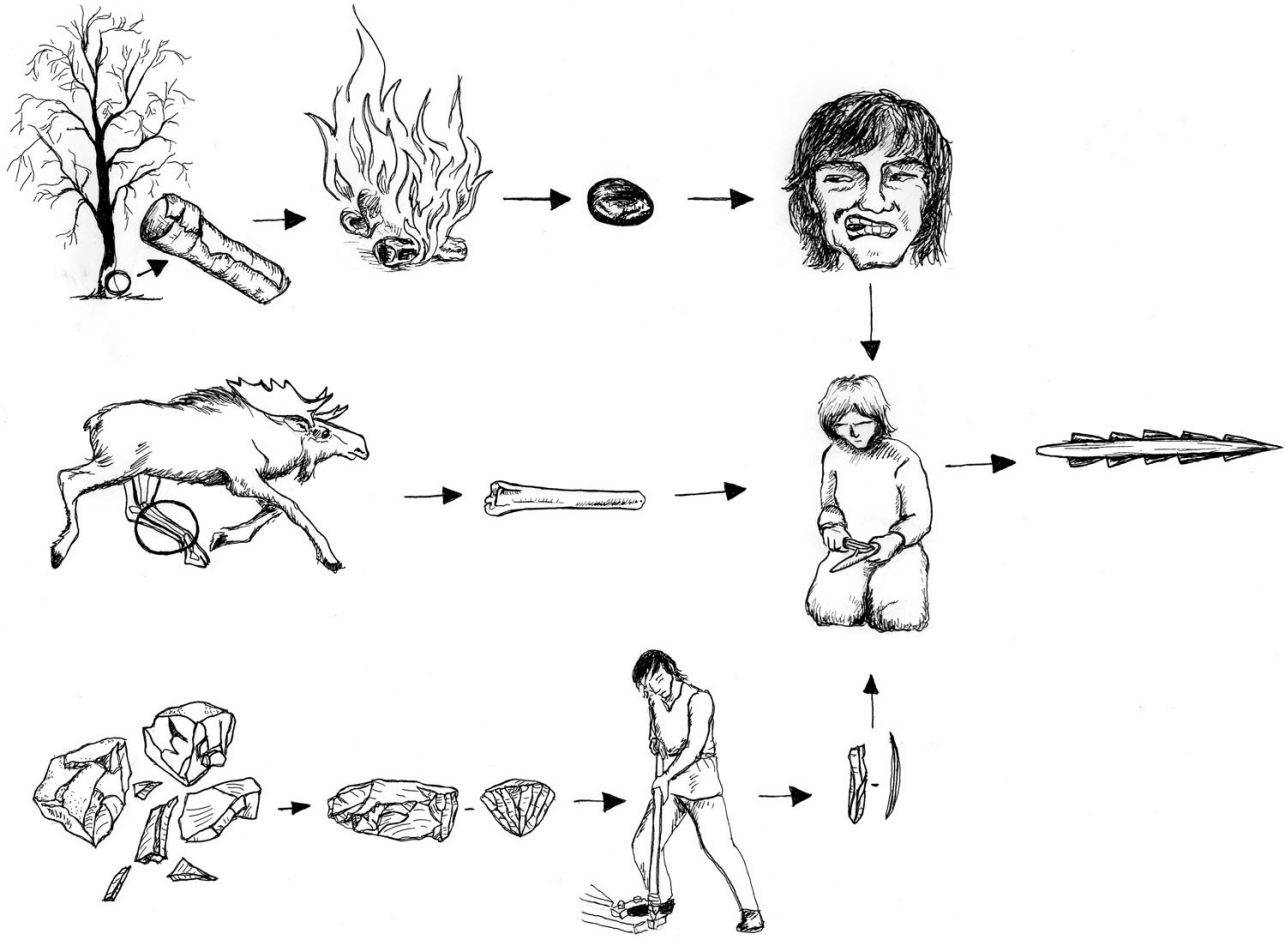
Operational chains used in the processing of raw materials during composite bone point production. Lithic blades served as inserts and birch bark pitch was used as an adhesive agent. Drawing: Kristina Steen^68^ (reproduced with permission from Universitetsforlaget Oslo)

In addtion, some standardised blade production and re-tooling was performed on-site, visible in the presence of discarded tools (a relatively low number) and regular blade blanks. Blades were produced by the same overall concept: serial production from single-platform, sub-conical and conical cores with faceted and smooth platforms. No complete regular blade cores are present, but fragments of conical cores with visible scars deriving from the detachment of very regular thin blades, along with core rejuvenation flakes with small-flake faceting and a platform to front angle close to 90°, suggest that the eastern pressure blade concept was employed (Supplementary Note 1, Supplementary Fig. 14). Although the majority of the studied blades display features found in blades produced by direct and indirect percussion techniques, a selection of blades display diagnostic characteristics of the pressure technique, and the variation in knapping techniques is best explained as related to the different stages of the production process (Supplementary Note 1). Morphometric analysis shows the production of a consistent range of blade blanks, which in turn allowed the production of standardised tools, such as barbed points (hulling-type), slender lanceolate microliths, as well as blades with lateral retouch on one or both edges. The last mentioned were probably used as inserts in composite slotted tools, to which the inserts were attached using mastic made of birch bark pitch^44^ (Fig. 5). A bone point with remains of pitch retrieved from the deep pit shows that birch bark pitch mastic was part of tool production at the site, while fragments of slotted points, contextually dated to the same period as the finds from the deep pit, were found nearby^45^.

## Discussion

Prior to our study, ancient DNA has been retrieved from biological remains of ancient individuals: bones, mummified tissue^46^, hair^47,48^, quids^49,50^ and coprolites^51^. Human aDNA in soil samples has also been discovered and processed, but so far only to determine the presence of homo species^52,53^. Our results from ancient mastic add to the available sources for genomic data on ancient individuals. DNA from saliva, preserved in the mastics, yields ancient human DNA, the authenticity of which we can confirm by studying damage patterns, contamination rates and population genomic analysis (Supplementary Fig. 1, Supplementary Table 5, Figs. 2 and 3). The genomic data from the mastics allows us to determine a close affinity between individuals from Huseby Klev and the previously defined SHG genetic group (Fig. 2). F4 and F3 tests confirm a close affinity to SHG (based on significant *Z*-score values for our best sequenced individual, ble008) (Fig. 3). The possible greater affinity to individuals found in Sweden (the Mesolithic SHGb subgroup) that we suggest here, should be further investigated. Using admixture analysis, we show that the studied Huseby Klev individuals have more of the WHG than the EGH components (Fig. 4), which is consistent with the genomic composition of the SHG individuals. The Huseby Klev individuals allow us to root the SHG group and extend its chronological span, as well as geographical distribution. Prior to this study, genetically defined SHG individuals either had no association with lithic technology (SHGa: Steigen and Hummervikholmen) or the associated artefacts were not sufficient to make inferences about stone tool production technology known to the buried individual (SHGb: Stora Bjers). The only two links established between genetics and lithic technology are found in the Stora Förvar cave’s Mesolithic layer (SHGb) and at the Motala Kanaljorden site (SHGb). The Motala Kanaljorden lithic inventory has characteristics typical of the handle core technology^54^, a blade production technology common in Late Mesolithic Scandinavia, and different from the eastern blade production technology^8,54^. In Stora Förvar, the lithic inventory has techno-typological characteristics of a technology typical to the first postglacial pioneers entering Scandinavia from the south, including blade production from single- and dual-platform cores by direct percussion techniques^55^. Table 2 shows the different lithic technological traditions, all found within the genetically defined SHGb group (as a context with SHGa and stone tools is currently lacking). The earliest documented technology (context dated to 10,040–9610 calBP) associated with SHG is the eastern blade technology visible in the production waste from Huseby Klev. This variety of technological traditions within the SHG group reminds us that genetic and cultural features are compatible only to some degree, and that we should be careful when merging information on cultural evolution and demographic processes.

**Table 2:** Scandinavian Mesolithic sites where both human aDNA and lithic artefacts are found

Site	Context date	Genetic group	Blade production technology
Huseby Klev	10,040–9610 calBP	SHGb	Eastern pressure blade technology
Stora Förvar	9170–8000 calBP	SHGb	Maglemosian technogroup 1 and 2
Motala, Kanaljorden	7760–7520 calBP	SHGb	Handle core technology

Context date is based on calibrated radiocarbon dates. Individual dates and the principle used in determining a context date are given in Supplementary Note 2 and Supplementary Excel Table 4. Genetic group refers to a subdivision of the SHG group into SHGa and SHGb

The results from Huseby Klev allow us to finally connect the SHG group with the eastern pressure blade technology. However, the genetic affinity between Huseby Klev individuals and the WHG group in comparison to the EHG group, challenges the earlier suggested tie between eastern technology and genetics. In the BLE individuals we discover less genetic affinity to EHG, and possibly to SHGa, thus we suggest either early cultural transmission, or a more complex course of events involving both non- and co-dependent cultural and genetic admixture. By combining genomic data and the archaeological dwelling site context, we are able to gain new insights into the Mesolithic society and discuss the social organisation of of past populations. The fact that each of the studied mastic pieces was chewed only by single individuals, both male and female, and that the mastication of birch bark pitch was most likely connected to the process of tool-making and maintenance (an interpretation supported by the evidence of core processing, re-tooling, and hunting found at Huseby-Klev), allows for a discussion of gender roles within Mesolithic society. Combined with the fact that several mastics from the site have imprints of deciduous teeth^11^, the new information allows us to discuss gender in childhood. The possible interpretations are that tool-production was not restricted to one sex, or, if the individuals examined were children, that gender roles did not yet apply to young individuals. When results from other dwelling sites besides Huseby Klev start to accumulate, we will be able to discuss the social organisation of past populations on a wider scale.

It is known that birch bark pitch mastics, but also mastics of other materials, have been widely used around the world from the Middle Palaeolithic onwards (Supplementary Note 1), including in regions where human remains are not available to study, either due to bad preservation (e.g., large parts of Fennoscandia), or restrictions for the use of human remains (such as the Kennewick Man conflict^56^). In these situations mastic pieces offer a possible source for DNA. In addition, mastics are expected to be a source of information concerning the environment, ecology and oral microbiome of prehistoric populations.

## Methods

### Mastics of birch bark pitch in Huseby Klev

A number of the Huseby Klev mastics bear traces of human teeth, while all of them have a chewing gum like morphology, a dark colour and a glossy surface. While modern experiments show that relatively simple methods can be used in the production of birch bark pitch (Supplementary Note 1), the production technology is somewhat knowledge intensive. It is mostly for this reason that teeth marks in the pitch are often considered indicative of processing and use, i.e., making the pitch more viscous and pliable, rather than a sign of purely recreational use as a chewing gum. It is known that birch bark pitch was used in hafting stone tools, and for attaching flint blades to slots in composite tools. Of the 115 finds of pitch from Huseby Klev (Supplementary Note 1), eight lumps from the Huseby Klev deep pit have been subjected to chemical analysis^57^. Seven of them turned out to be birch bark pitch while one did not give results. Alexandersen^11^ has studied ten lumps with tooth impressions and, by comparing to modern parallels of tooth development and wear, determined the age of the chewers in these cases to have been between 5 and 18 years. In addition, a piece with teeth impressions from both an adult and a child has been reported^58^. Other pieces of pitch from the deep pit show a variety of wood and cordage impressions^59^.

### Sample preparation

We chose eight birch bark pitch mastic pieces for analysis. After the first screening, we continued working with three of the samples: *ble004* (no artefact number assigned), *ble007* (artefact number: Fnr. 8031 Gr 9) and *ble008* (artefact number: Fnr. 8723 B Gr 9). None of our samples had teeth imprints, but had clear morphological similarities to chewed bubble gum. The samples were processed in the clean room facilities of the Archaeological Research Laboratory (AFL, Stockholm University). The facilities are reserved for ancient DNA work, and access is restricted and used only by professional personnel. There are airlocks by the entrance to the clean laboratories, which have positive pressure and installed HEPA filters. While working, employees wear protective suits, visors and gloves. Surfaces are frequently processed with sodium hypochlorite based solution and UV lighting is used to insure cleanliness. The mastic pieces were irradiated in a cross-linker, at about 6 J/cm^2^ at 254 nm. The outer shell of the mastics was discarded to avoid surface contaminants. The powder for extraction was produced using a Dremel drill or a scalpel and collected into 2 ml tubes. The weight of the obtained powder varied between 66 and 191 mg.

### Extraction

To extract the DNA, we performed several incubations on all of our samples. At each incubation the samples were kept in rotation. First, the samples were pre-digested at 45 °C for 15 min in 1000 μL of extraction buffer, consisting of Urea, EDTA (0.5 M) (VWR) and 10 μL of Proteinase K (10 mg/mL) (VWR)^30^. A negative control was added during this step and taken through the work process. The supernatant from the predigestion step was removed and a fresh extraction buffer (same as above) with proteinase was added to the samples and left for digestion overnight at 37 °C. The supernatant from this step was stored, and more extraction buffer and proteinase were added to the samples (same as above) and left rotating at 55 °C for 4 h. The final supernatant was collected and combined with the previous one (around 2000 μL) and spun down to 100 μL using membrane filters (Amicon Ultra-4 Centrifugal Filter Unit with Ultracel-30 from Millipore). The extract was purified using MinElute spin columns and a buffer set (both Qiagen). We modified the Qiagen protocol (reducing the PE buffer volume to 600 μL and performing two elutions using 55 μL of the EB buffer) and obtained about 110 μL of extract, which was stored at −20 °C. For ble004 sample two extracts were made from two different samplings. We performed an extraction on ble008 sample using QIAamp PowerFecal DNA Kit (Qiagen), which is used to remove inhibitors in stool, gut and biosolid samples. We used 0.25 g of the sample and collected it directly into a Dry Bead Tube, containing garnet beads, which during vortexing mechanically disrupts cell walls. We added a negative control at the first extraction step. After adding the reagents according to the protocol and performing the first incubation step, we used a thermoshaker and vortexed the samples at 65 °C for 30 min at the highest speed available. This was a necessary alteration, as we did not have the suggested machinery at our disposal. All the buffers were added according to the protocol provided with the kit, except during step 14, where we reduced the amount of C4 solution to 1000 μL. As the extraction was finished, the DNA got captured on a silica membrane of a spin column and purified, resulting in 100 μL of product which was stored at −20 °C.

### Libraries and sequencing

We built double-stranded blunt-end libraries, using the modified protocol by Meyer and Kircher^31^. We used 20 μL of the extract to build the blunt-end repair libraries and 30 μL for the USER enzyme pre-treated libraries. The master mix for the blunt end repair step contained 4 μL of Buffer Tango 10× with BSA (Thermo Scientific), 0.16 μL 25 mM dNTP mix (Thermo Scientific), 0.4 μL ATP 100 mM 25 μmol (Thermo Scientific), 12.64 μL ddH_2_O, 2 μl T4 Polynucleotide Kinase (10 U/μL) (Thermo Scientific) and 0.8 T4 DNA Polymerase (5 U/μL) (Thermo Scientific), and incubation for this step was for 15 min at 25 °C, followed by 5 min at 12 °C. We used MinElute spin columns to purify the product, reducing the proposed volume of the washing buffer PE to 600 μL. The ligation master mix contained 10 μL ddH_2_O, 4 μL 10× T4 DNA ligase buffer (Thermo Scientific), 4 μL PEG4000 50% (w/v) (Thermo Scientific), 1 μL of Adaptor mix P5/P7 100 mM (10 pmol) (Biomers.net) and 1 μL of T4 DNA ligase 5 weiss U/μL (Thermo Scientific), and was incubated at 22 °C for 30 min. We purified the product as above and continued to fill in the adaptors with the following master mix: 14.1 μL of ddH_2_O, 4 μL of 10× ThermoPol reaction Buffer (BioLabs), 0.4 μL of 25 mM dNTP mix (Thermo Scientific) and 1.5 μL Bst DNA Polymerase Large Fragments 8000 U/mL (BioLabs). The incubation steps were 37 °C for 20 min, followed by 20 min at 80 °C. We performed UDG treatment before blunt-end repair for several libraries^60^. We used USER Enzyme 1000 U/mL (BioLabs) and incubated the extract with the ingredients for a blunt-end mastermix (excluding polymerase, which was added after the incubation) for 3 h at 37 °C. We then proceeded with the blunt-end protocol. For sample ble004 nine libraries were built, five of which have been pretreated by USER enzyme. For ble007 sample five libraries were produced, for ble008 sample six libraries. Libraries were amplified with 10 µM index primers (Biomers.net), using AmpliTaq Gold 1000 Units 5 U/μL (Applied Biosystems) for blunt-end libraries, and AccuPrime^™^ Pfx DNA Polymerase (2.5 U/μL) (Invitrogen) polymerase for damage repair libraries. We determined the number of cycles using quantitative PCR, with reagents from Thermo Scientific and Biomers. Libraries were sequenced on the Illumina Hiseq X platform at the SciLife centre in Stockholm.

### aDNA investigation and data processing

We merged paired-end reads if they had at least 11 nucleotides of overlap using the *MergeReadsFastQ_cc.py* script^61^ and processed reads for any remaining adaptor sequences. Afterwards, we treated each sequence as single-end read and aligned to human reference with custom parameters (*bwa aln* command with seeds disabled -l 16500 -n 0.01 -o 2) to allow more mismatches and gap events^34,39,61^. We merged mapped libraries (*ble004_dr*, *ble004_nondr*, *ble007*, *ble008 and ble008 new method*) using *samtools merge*, and filtered reads that are PCR duplicates with *FilterUniqSAMCons_cc.py* script, having less than 90% sequence identity with the reference chromosome, smaller than 35 nucleotides, and mapping quality less than 30^61^. For *ble004*, we merged filtered damage repaired and non-damage repaired libraries to produce the final bam file.

### Principal component analysis

For this analysis, we merged the libraries of ancient individuals with Human Origins dataset separately by coding the nucleotide transitions as missing data. We used the *smartpca* software to calculate eigenvalues for each ancient individual. Then we used Procrustes transformation to project ancient individuals on the principal component space. Human origins reference genome set contains 594,924 SNP’s from 2404 modern individuals from 203 populations worldwide^39,40^. To compensate for the biases that could be introduced from PMD decay, we coded deamination transitions as missing data. PC plot with the entire Human Origins database is shown in Supplementary Fig. 3.

### F4 and F3 statistics

To test the population affinities between the ancient individuals, we used the *popstats* programme to calculate F4 and F3 statistics^62^. These tests propose formal statistical frameworks to study the patterns of allele frequency correlation across populations^40^. F4 test provides evidence of significant deviations from a tree-like population structure, which could be demonstrated as ((A, B) (X, Y)). Positive-F4 values indicate a population affinity between A, X and B, Y. Moreover, F4 tests give information about the direction of the shared genetic drift. Similarly, positive values indicate a shared genetic drift between A and X and B and Y. In both cases, negative values indicate a relationship between A, Y, and B, X. F3 test analyses the shared genetic drift between two populations or individuals (denoted as A and B) from an outgroup (C) as ((A, B) (C)). We followed the workflow as described in Skoglund et al.^62^: we computed standard errors using a block jackknife weighted by the number of SNPs in each 5 cm and we reported *Z*-scores as normalised *Z* = *D*/s.e. *Z* > 2 was interpreted as a significant deviation from zero. We used the flag –f4 as described in the manual.

### Model-based clustering

We used a model-based clustering algorithm called *admixture* to understand the population structure in our dataset^42^. To use our merged data with this tool, we first pseudo-haploidized the dataset by removing one allele randomly from the reference panel. Then we filtered our dataset for linkage disequilibrium using Plink with the parameters*–indep-pairwise 50 5 0.5*^63^. We ran *K* = 2 to *K* = 20 with 20 different replicates using different random seeds. To detect common signals observed in independent Admixture runs, we used a greedy algorithm implemented in *pong* software^64^ (Supplementary Fig. PDF 1, Supplementary Fig. PDF 2).

### Lithic technology

The lithic blade production concept at Huseby Klev was reconstructed by defining the production methods and knapping techniques used at the site. A dynamic-technological classification including a simplified *chaîne opératorie* analysis of the complete lithic assemblage and an attribute classification of a selection of the artefacts was employed as the methodological basis (Supplementary Note 1, Supplementary Fig. 13)^65–67^. In all 1849 flint artefacts from the deep pit were studied. Altogether 86 artefacts were considered high priority in determining blade production methods and knapping techniques, and were subsequently selected and catalogued according to the attribute classification (Supplementary Note 1).

### Reporting summary

Further information on experimental design is available in the Nature Research Reporting Summary linked to this article.

## Supplementary information

Supplementary Information

Reporting Summary

Supplementary Data 1

Supplementary Data 2

Supplementary Data 3

Description of Additional Supplementary Files

## Data Availability

The sequences are available at the European Nucleotide Archive (ENA) with the accession number PRJEB30667. The Supplementary PDF and Excel Tables data has been deposited in Dryad Digital Repository: 10.5061/dryad.j3k19p9.
