# Supplementary material for: Ancient DNA from mastics solidifies connection between material culture and genetics of mesolithic hunter–gatherers in Scandinavia
Source: Commun Biol. 2019 May 15;2:185. doi: 10.1038/s42003-019-0399-1 (PMC6520363; doi:10.1038/s42003-019-0399-1)
Supplement: Supplementary file 1 — Supplementary Information [file 42003_2019_399_MOESM1_ESM.docx]

**SUPPLEMENTARY INFORMATION**

# Supplementary figures


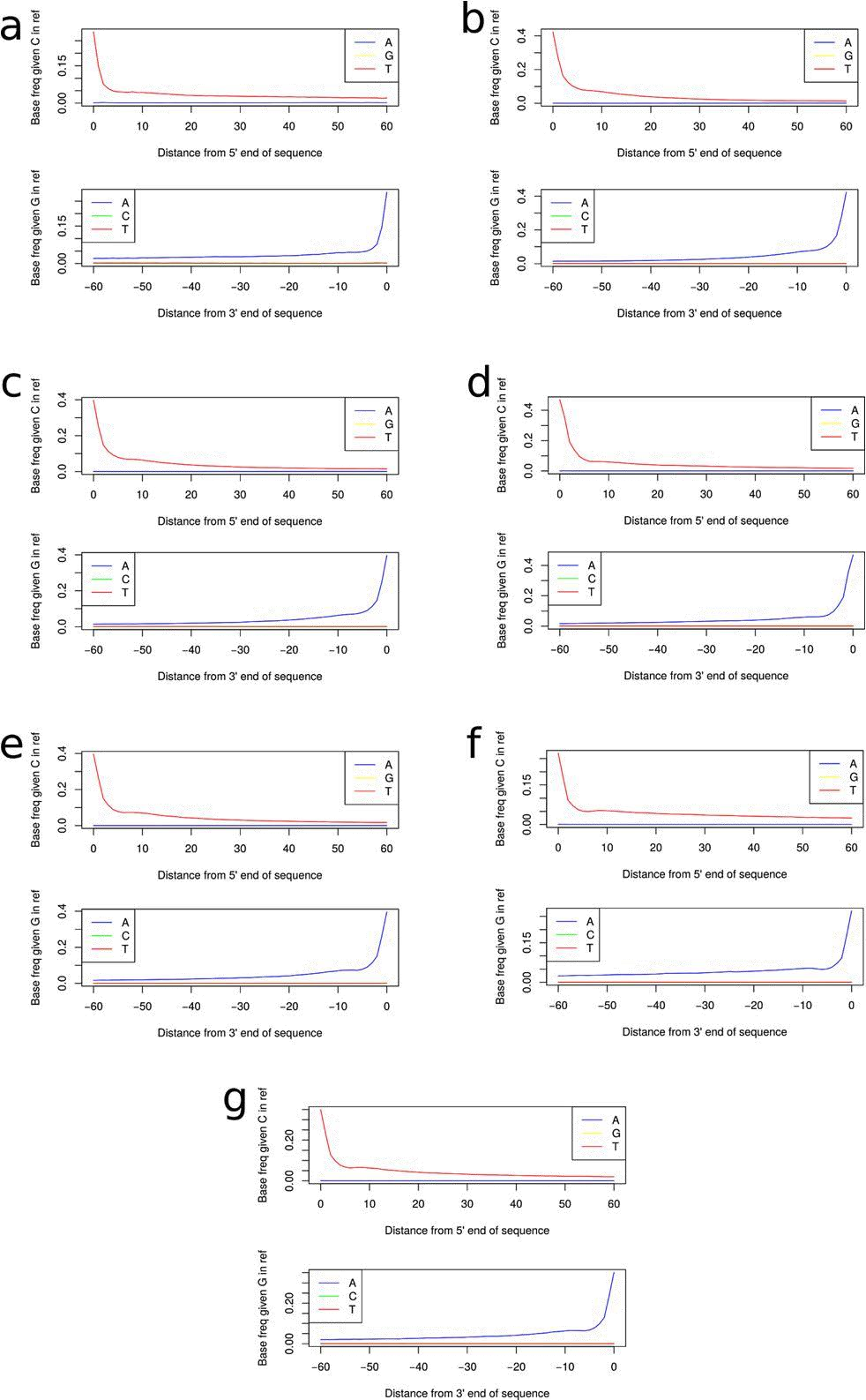


**Supplementary Figure 1**. Deamination patterns observed in libraries. Ble004dr (a), ble004_nondr(b), ble004_merged (c), ble007 (d) ble008 (e) ble008_newmethod (f),

ble008_merged (g)


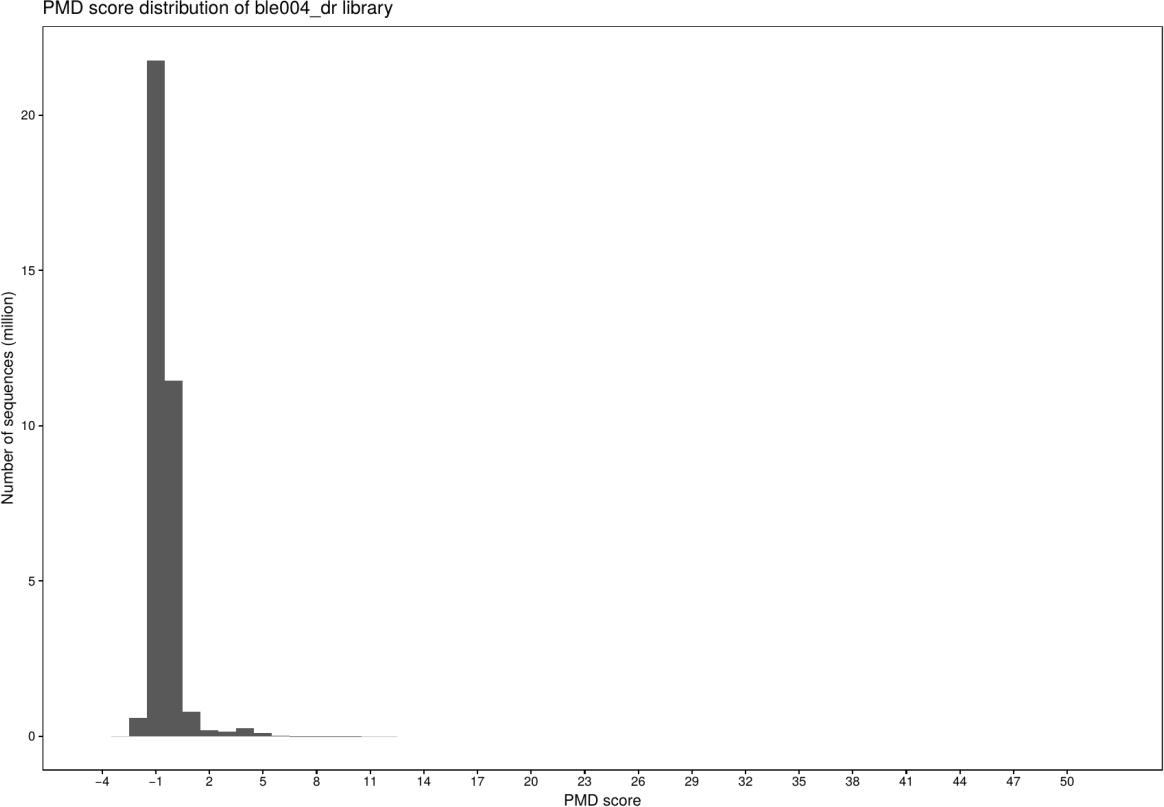


**Supplementary Figure 2**. PMD score distribution observed in libraries


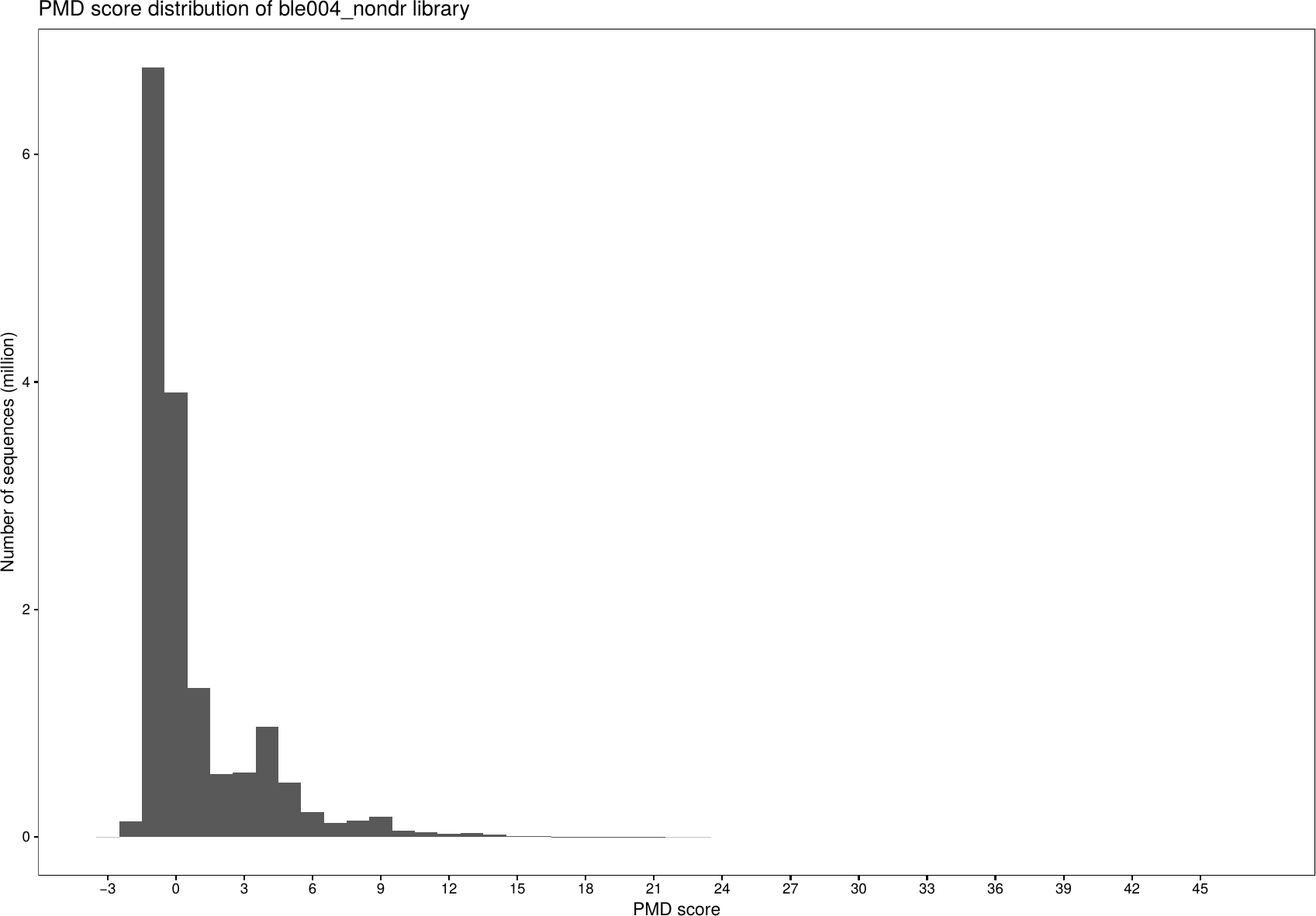


**Supplementary Figure 3**. PMD score distribution observed in libraries


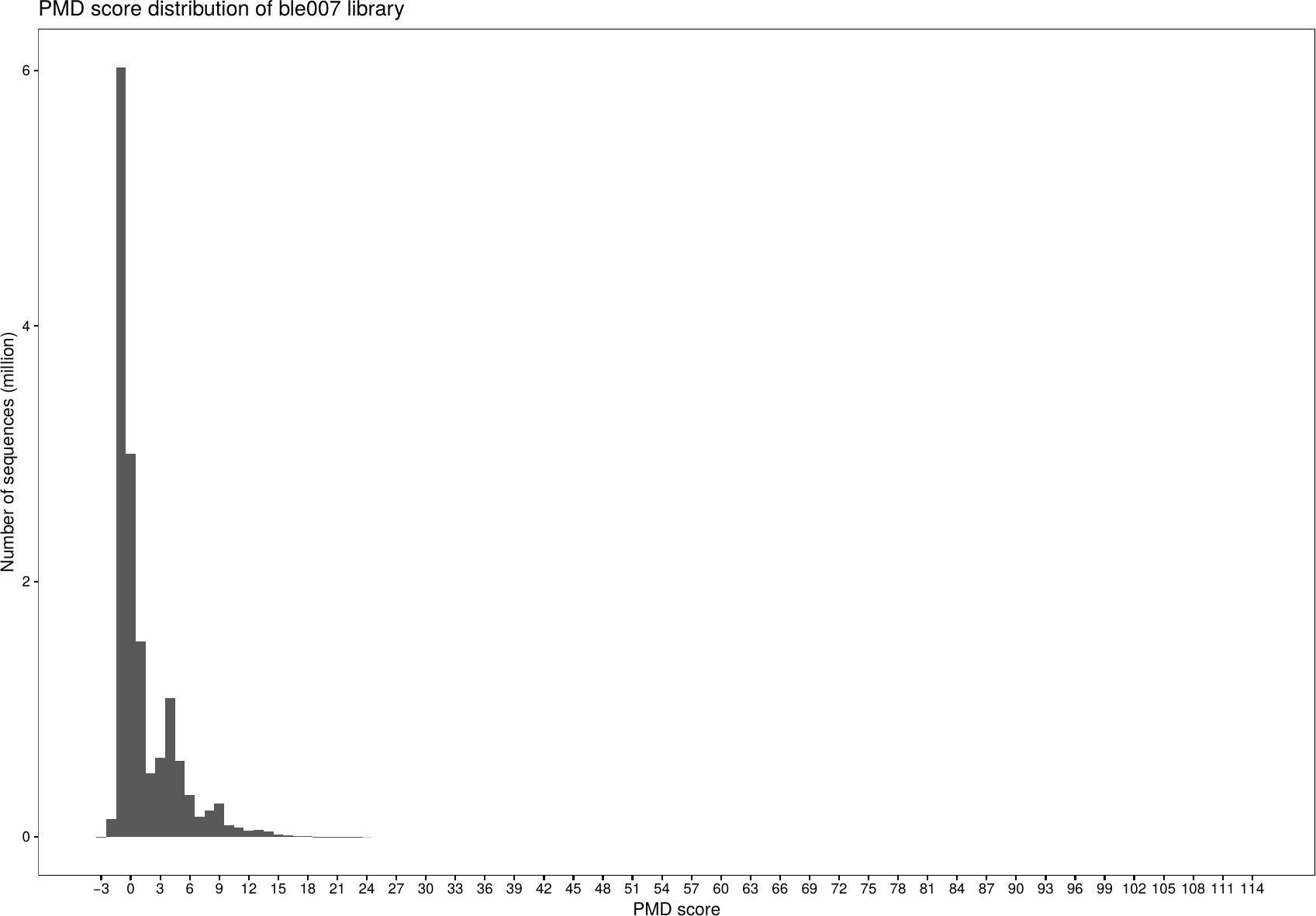


**Supplementary Figure 4**. PMD score distribution observed in libraries


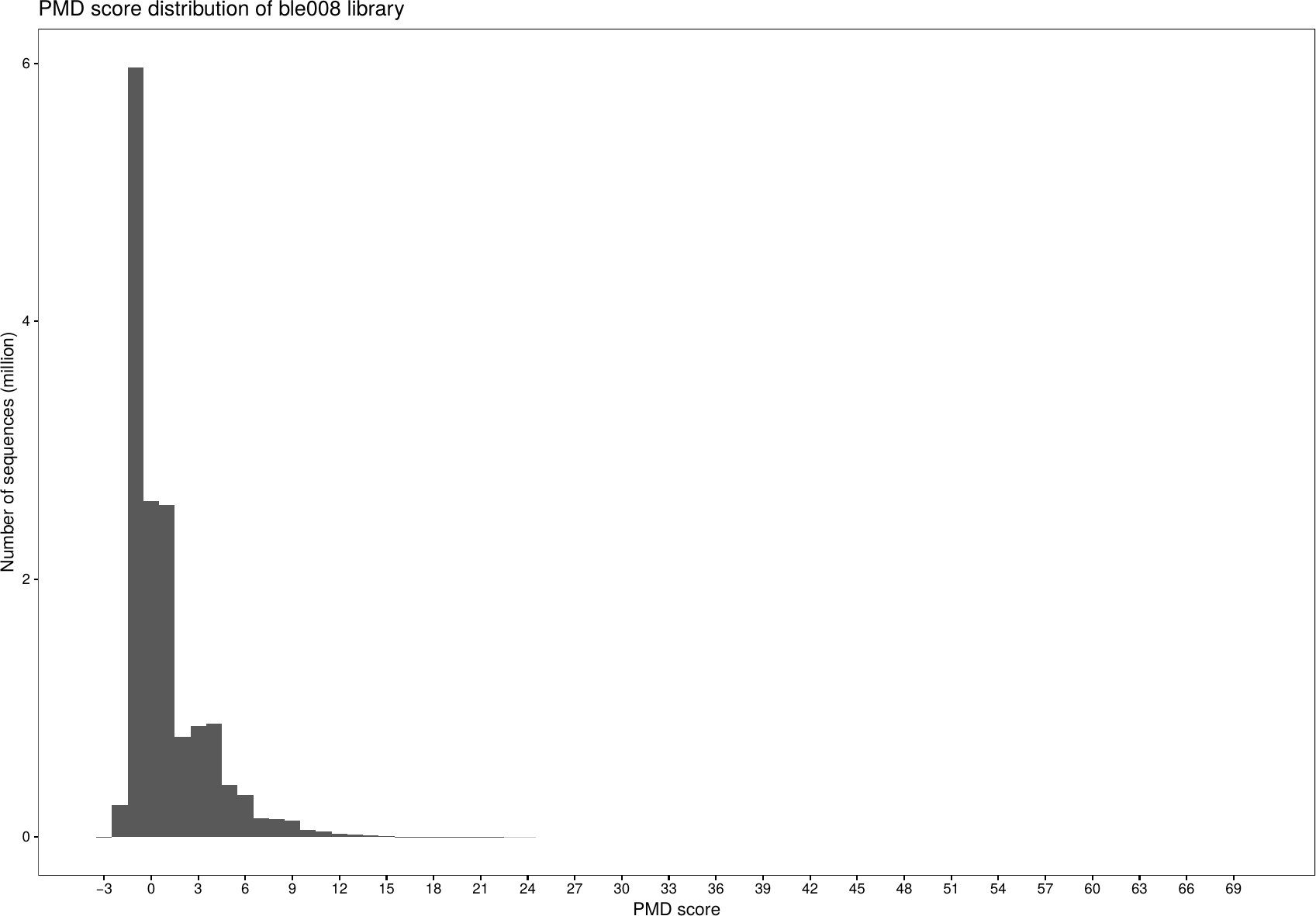


**Supplementary Figure 5**. PMD score distribution observed in libraries


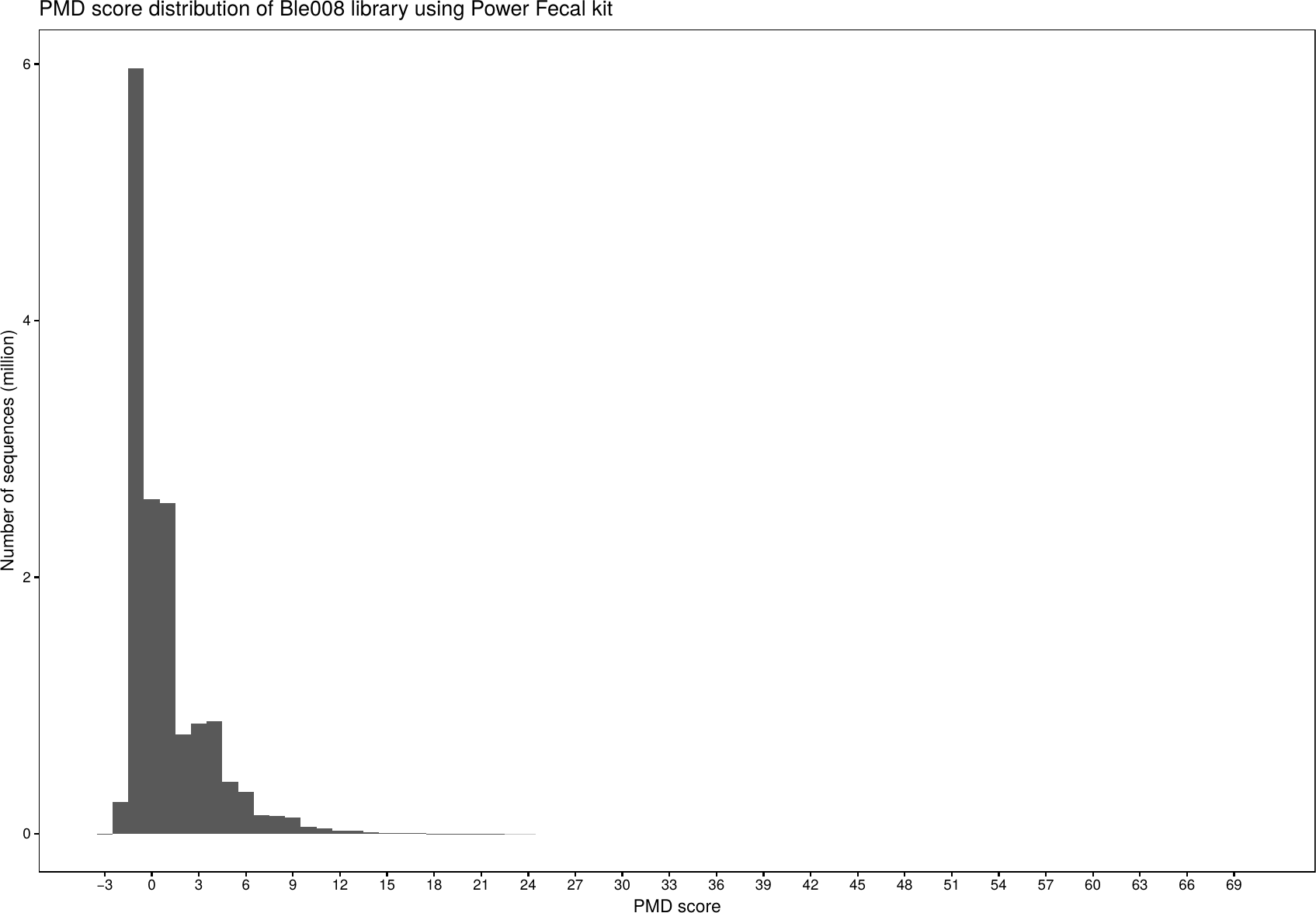


**Supplementary Figure 6**. PMD score distribution observed in libraries


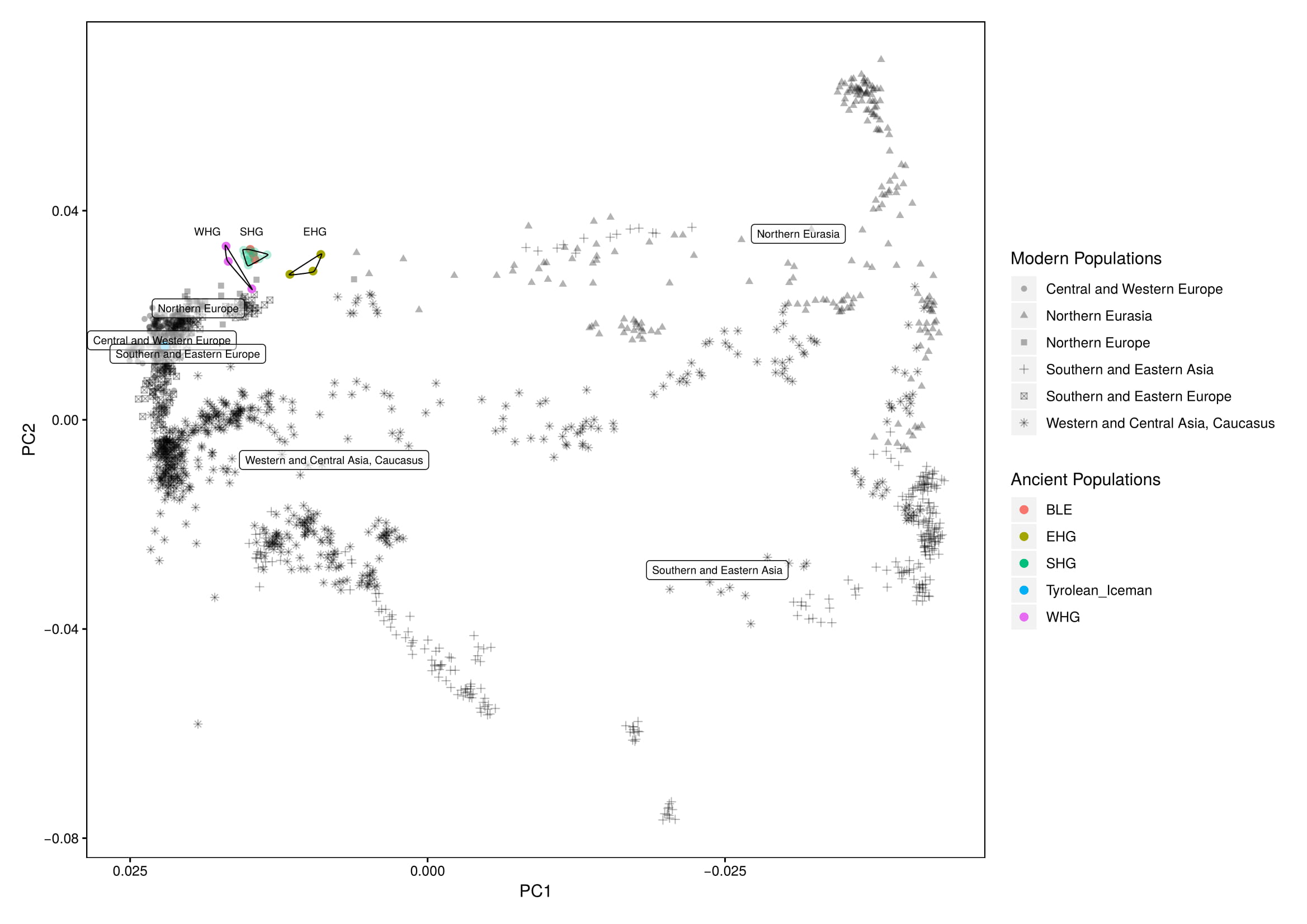


**Supplementary Figure 7**. Principal component analysis of the Huseby Klev individuals along with SHG, EHG, and WHG groups.


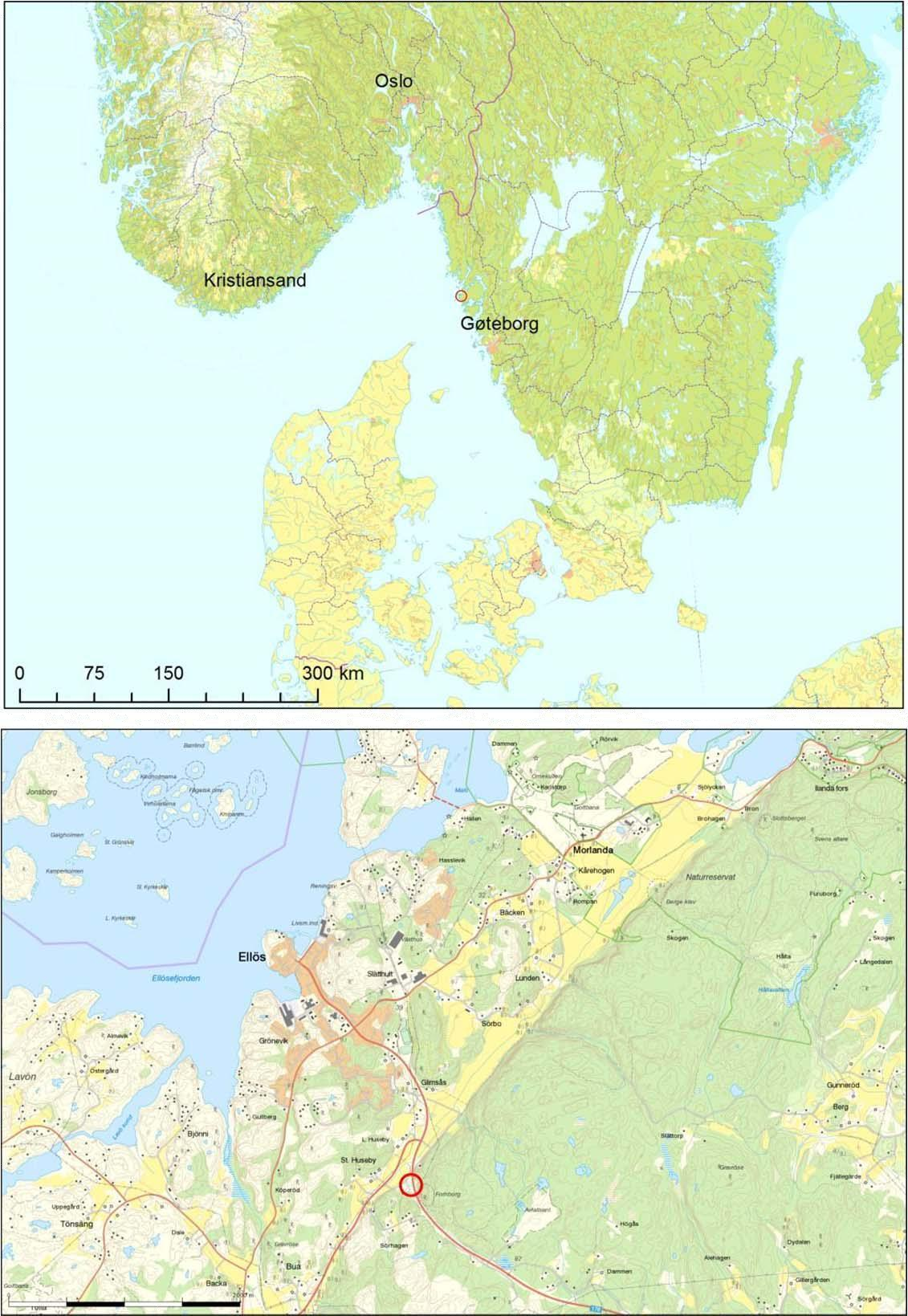


**Supplementary Figure 8.** Maps showing the location of the Huseby Klev site, marked with a red circle.


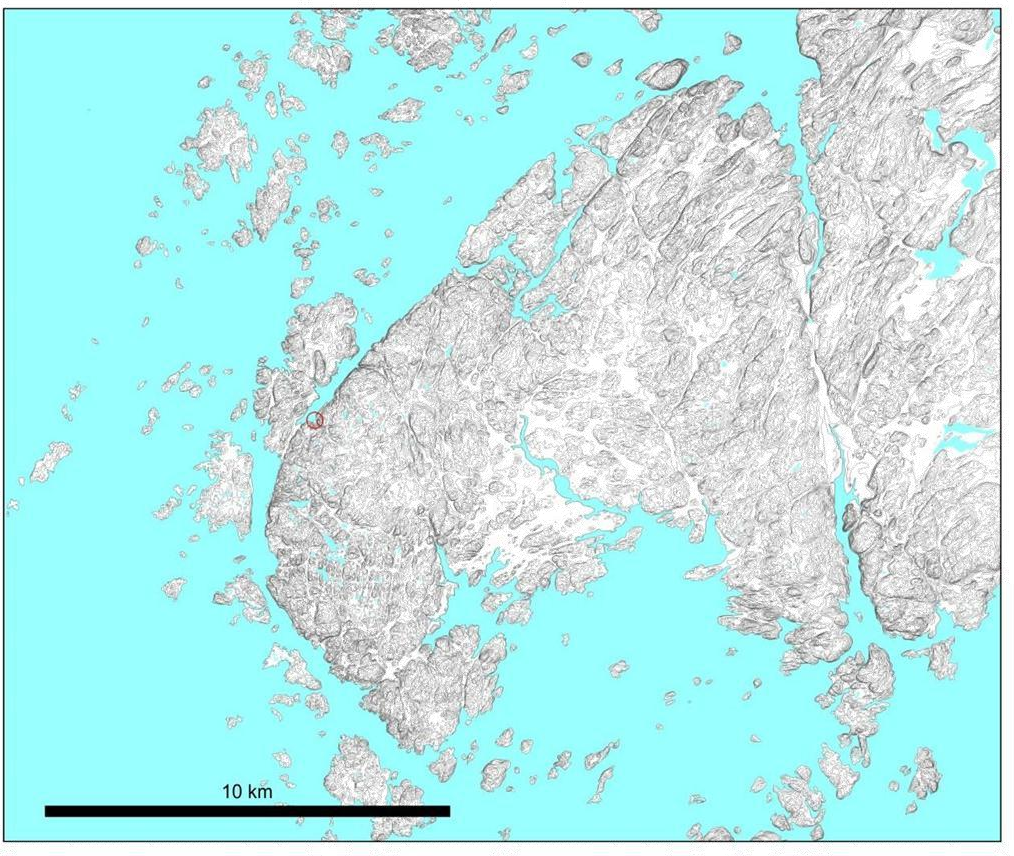


**Supplementary Figure 9.** The Huseby Klev site with a shoreline 25 meters above present sea level, illustrating the local topography during the period from which the finds in the deep pit trench originate.


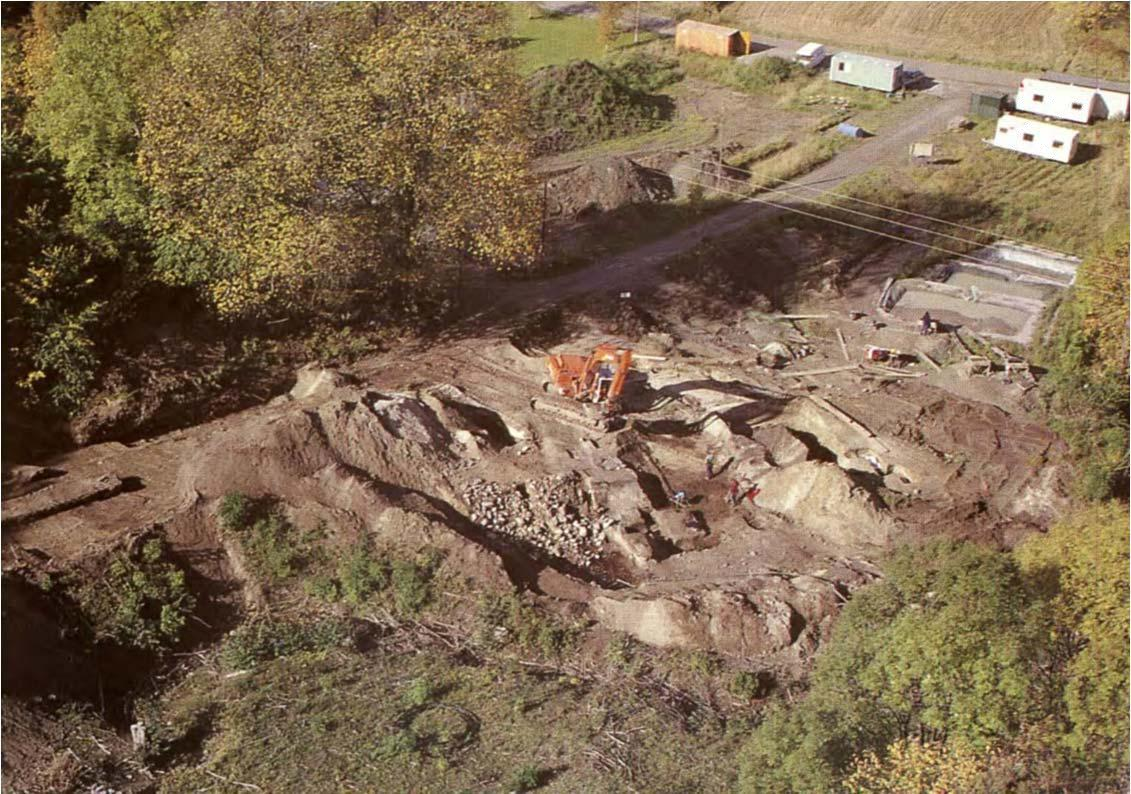


**Supplementary Figure 10.** The Huseby Klev site during the excavation in 1993. Photo towards NW. At the time when this photo was taken the excavation of the deep pit had not yet begun. The deep pit was later dug in the area in which the heap of soil is situated in the upper middle part of the photo. Photo: Robert Hernek.


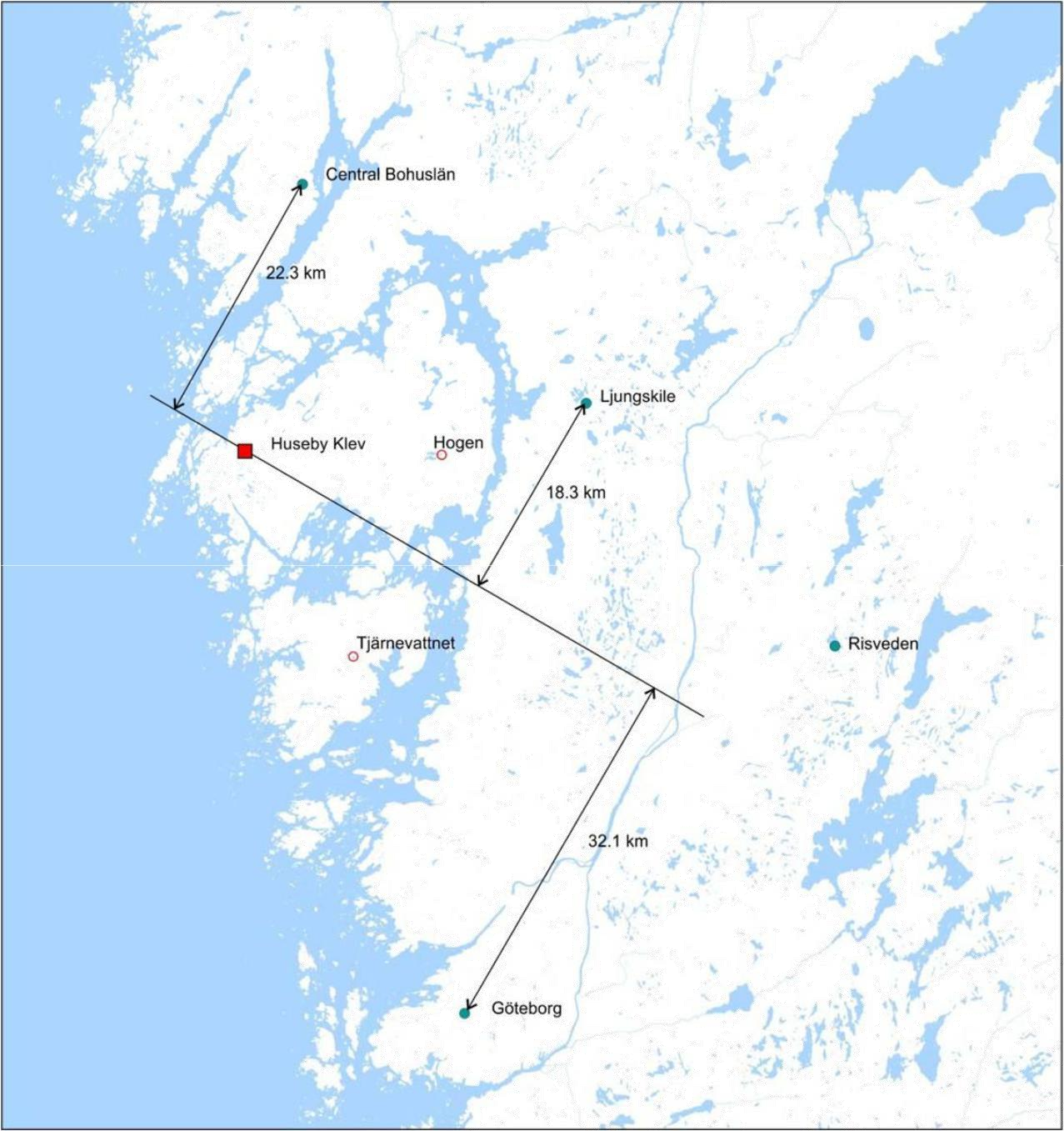


**Supplementary Figure 11.** Southwestern Sweden with Huseby Klev, marked with a red square, and the nearest locations with published shore line displacement curves. The line through Huseby Klev is perpendicular to N-30 degrees E, a bearing that connects places with the same shoreline displacement rate in this region. The references for the curves are: Central Bohuslän^38^, Ljungskile^36^, Risveden^39^, Göteborg ^37^. Hogen and Tjärnevattnet are places where the height of the transgression maximum has been determined by Persson ^36^.


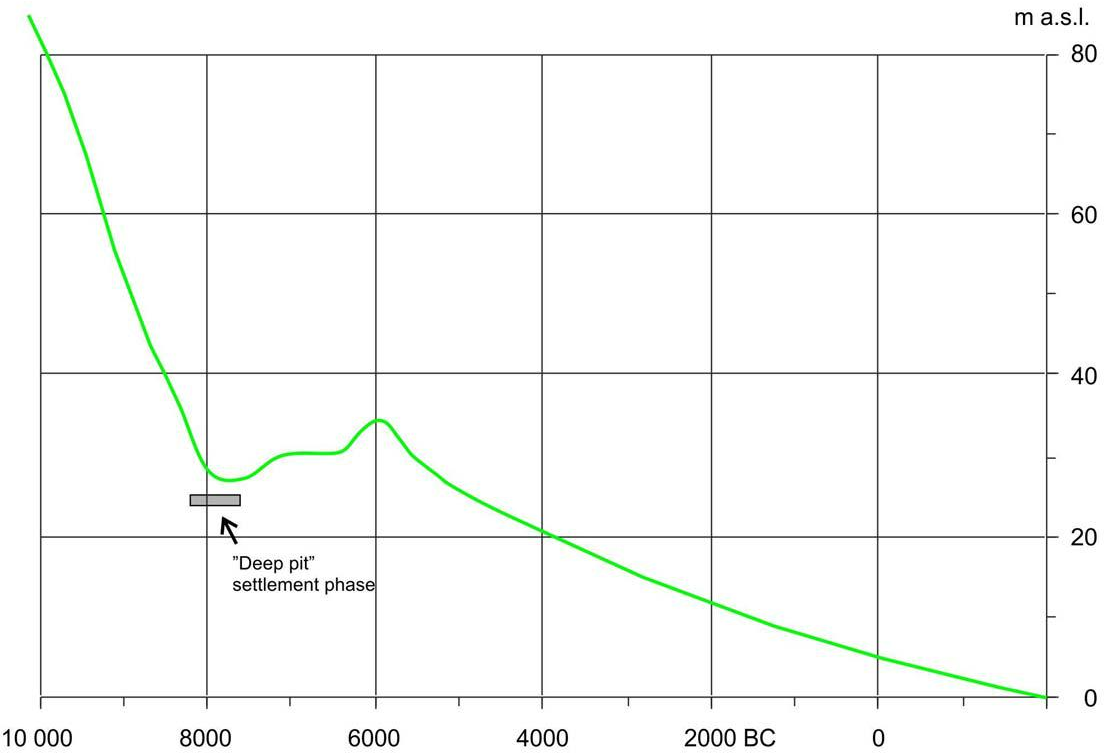


**Supplementary Figure 12.** Tentative shore line displacement curve for Huseby Klev. Based on the studies conducted in the locations shown in Figure 4.


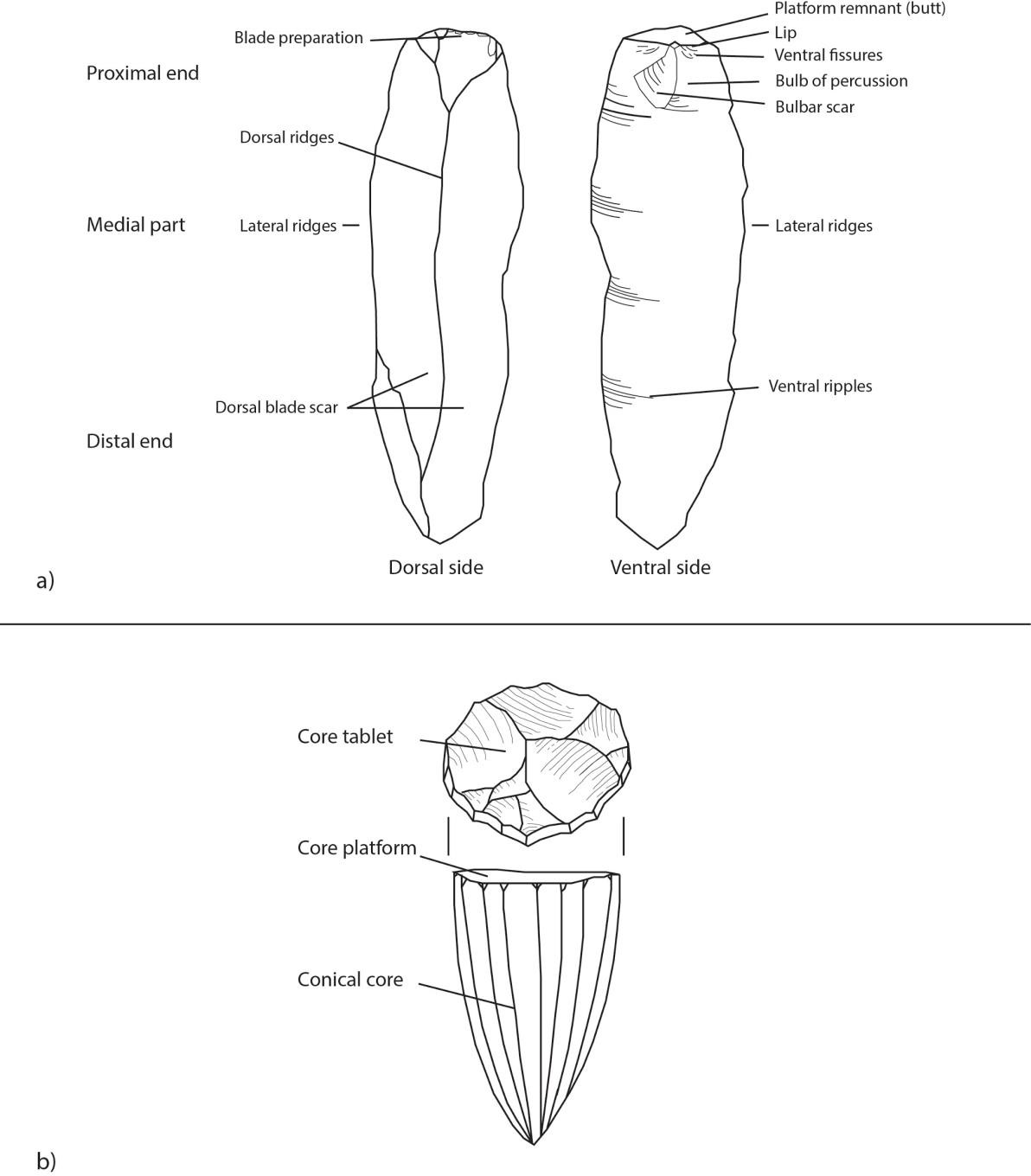


**Supplementary Figure 13.** Schematic figure illustrating central a) blade- and b) core attributes mentioned in the text ^2,^ ^27^. Illustration: H. Damlien.


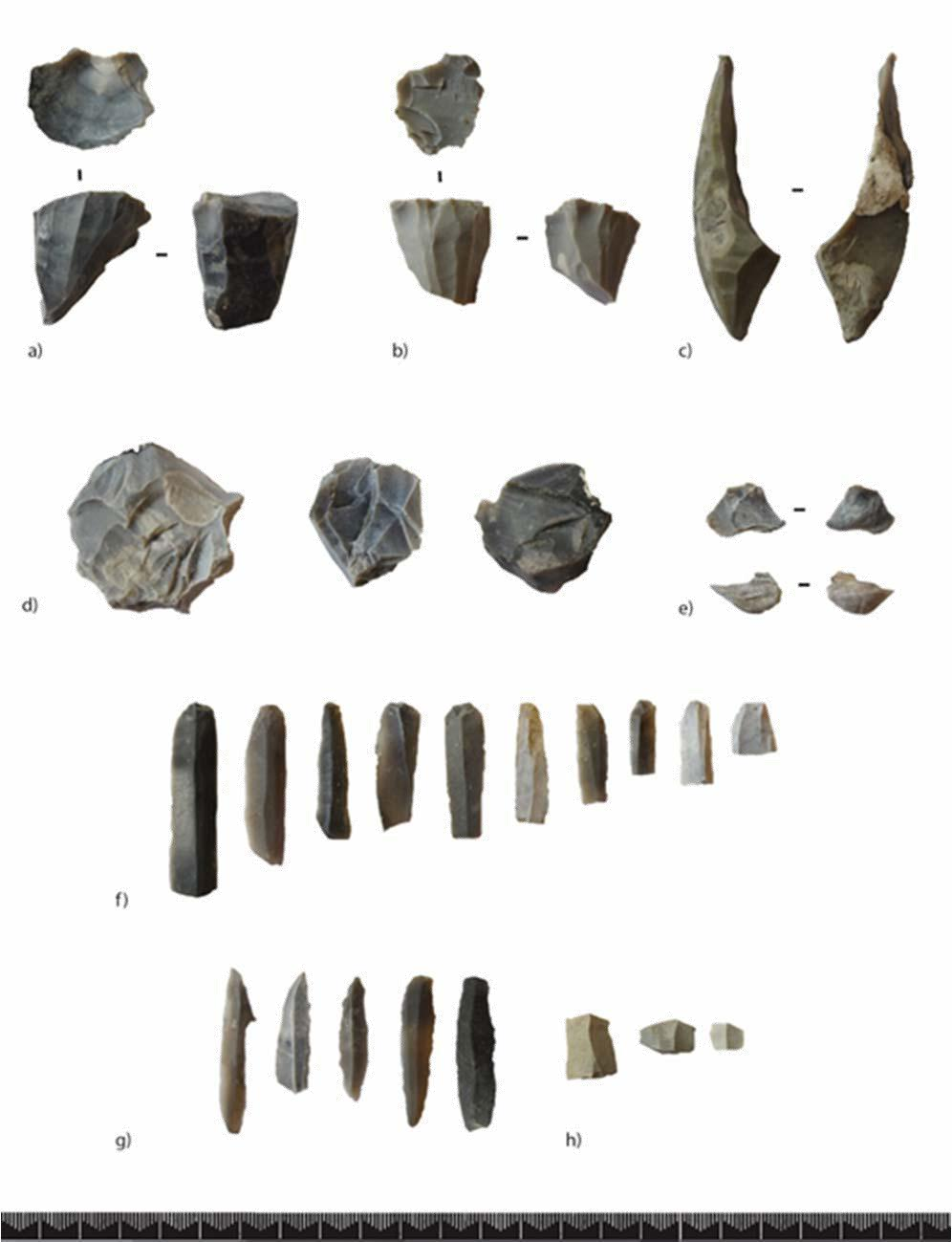


**Supplementary Figure 14.** A selection of artefacts from the deep pit of Huseby Klev considered diagnostic to the eastern pressure blade technology: a-c) fragments of conical cores with negatives after very regular blades, d) platform rejuvenation flakes (core tablets) with systematic faceting, e) small hinged preparation flakes, f) regular to very regular blades, g) barbed points, lancet microliths, bore and blade with lateral retouch, h) medial blade fragments. Photo: H. Damlien.


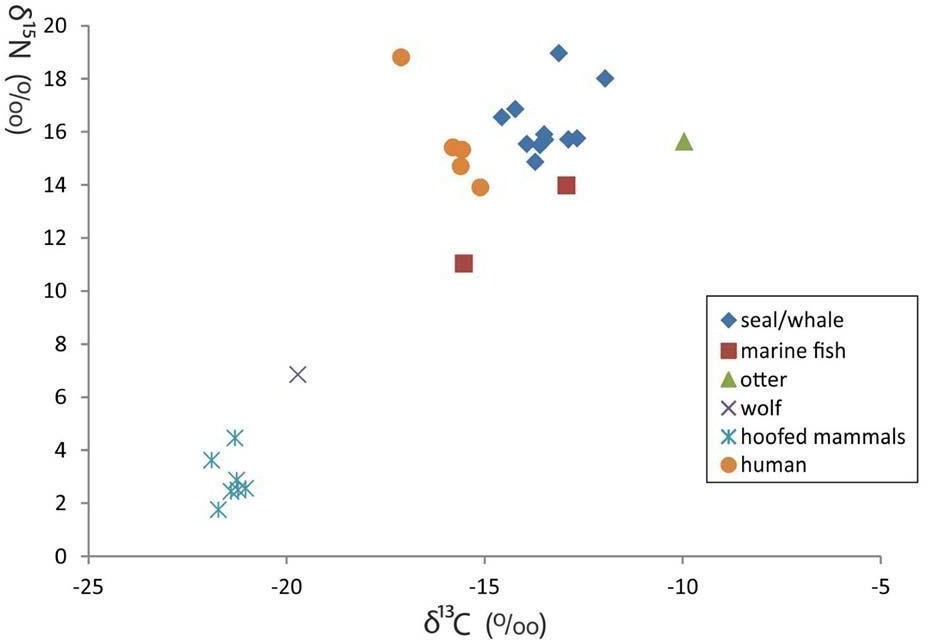


**Supplementary Figure 15.** Stable isotope values in bones found in the deep pit. Human bones group close to whale and seal, indicating that the Mesolithic inhabitants of Huseby Klev had a highly marine diet ^34,61^.


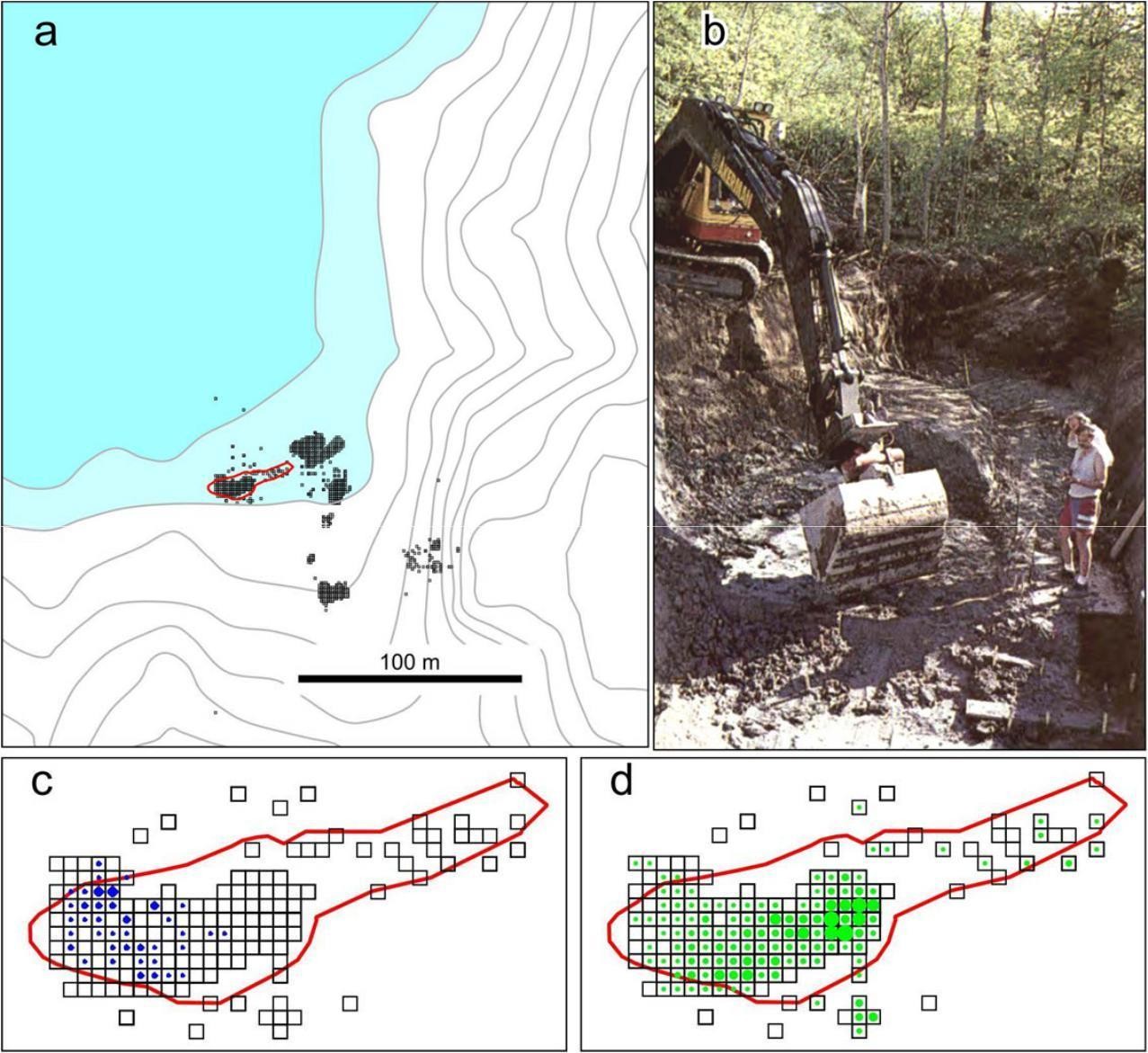


**Supplementary Figure 16. a.** The Huseby Klev site with 5 meter contour lines and excavated areas. The deep pit trench is marked with red. The areas below 20 and 25 m above sea level marked with shades of blue. **b.** Excavation of the deep pit in 1993. Bengt Nordqvist and Karl-Göran Sjögren are standing on the find-layer while the digging machine removes the overlying transgression layers. **c.** The distribution of mastics pieces, and d/ flint artifacts, with a maximum of 9 and 81 artefacts per square meter, respectively.

# Supplementary tables

**Supplementary Table 1.** Sequencing statistics for BLE samples. Data in this table comes from the best sequencing runs. Statistics for BLE merged samples is for all merged libraries, made from all produced extracts.

| Sample name and information | Merged sequences | Human sequences | Endogenous DNA | Average read length | Too short | Genome coverage | MT  coverag e |
| --- | --- | --- | --- | --- | --- | --- | --- |
| ble004 | 145,439,012 | 11,286,813 | 0.082392268 | 61.4431 | 5.582577057 | 0.175447 | 17.2384 |
| ble007 | 183,104,955 | 3,040,873 | 0.034630592 | 75.5933 | 4.723820236 | 0.0463599 | 3.31589 |
| ble008 (library built on Urea- Yang Extract) | 69,846,066 | 2,104,529 | 0.03094117 | 78.6352 | 2.637244872 | 0.0437531 | 2.41234 |
| ble008 (library built on Power Fecal Extract) | 71,984,716 | 15,236,588 | 0.2376068692  1373697 | 107.936 | 1.37791237087  93635 | 0.454355 | 17.2948 |

**Supplementary Table 2.** Library statistics for merged BLE libraries before filtering

| Sample | Human Sequences | Read length | Genome coverage | MT  coverage | MT  reads | X seq | Y seq | Sex |
| --- | --- | --- | --- | --- | --- | --- | --- | --- |
| ble004_no ndr | 15,570,111 | 65.00 | 0.24 | 25.39 | 10,671 | 705,576 | 46,095 | XX |
| ble004_dr | 35,344,643 | 55.00 | 0.50 | 53.79 | 24,007 | 1,586,07  0 | 107,291 | XX |
| ble004_m erged | 50,914,754 | 78.74 | 0.74 | 78.18 | 34,678 | 2,291,64  6 | 153,386 | XX |
| ble007 | 15,570,111 | 73.75 | 0.24 | 16.50 | 5,646 | 502,414 | 162,543 | XY |
| ble008 | 29,979,007 | 76.60 | 0.60 | 30.90 | 9,265 | 1,430,40  5 | 56,513 | XX |
| ble008_ne w_method | 15,236,588 | 105.15 | 0.45 | 17.29 | 3,555 | 756,905 | 20,068 | XX |
| ble008_m erged | 45,215,595 | 84.687  9 | 1.05674 | 48.1202 | 12,820 | 2,187,31  0 | 76,581 | XX |

**Supplementary Table 3.** Library statistics of merged BLE libraries after filtering

| Sample | Human Sequences | Read length | Genome coverage | MT  coverage | MT reads | X seq | Y seq | Sex |
| --- | --- | --- | --- | --- | --- | --- | --- | --- |
| ble004_ nondr | 4,872,698 | 68.2 | 0.08 | 10.11 | 3,716 | 210,087 | 13,775 | XX |
| ble004_ dr | 1,591,195 | 63.12 | 0.02 | 3.8 | 1,229 | 51,736 | 83,183 | Consis tent with XX but not XY |
| ble004_mer ged | 6,463,893 | 66.95 | 0.11 | 13.91 | 4,945 | 261,823 | 22,09  3 | XX |
| ble007 | 5,802,458 | 76.98 | 0.10 | 8.05 | 2,601 | 170,651 | 69,025 | XY |
| ble008 | 11,540,944 | 81.88 | 0.26 | 13.06 | 3,672 | 536,088 | 17,96  4 | XX |
| ble008_new  _method | 6,852,692 | 118.63 | 0.23247 | 10.1607 | 1,828 | 330,326 | 8,087 | XX |
| ble008_mer ged | 18,393,636 | 95.57 | 0.49 | 23.22 | 5,500 | 866,414 | 26,051 | XX |

**Supplementary Table 4.** Mitochondrial contamination estimates before filtering of BLE samples

| Sample | Point est | Informative | Consensus | total | Lower CI | Higher CI |
| --- | --- | --- | --- | --- | --- | --- |
|  | (%) | sites | sites | alleles |  |  |
| ble004_nondr | 13.30 | 5 | 176 | 203 | 8.63 | 17.97 |
| ble004_dr | 13.6 | 5 | 360 | 417 | 10.37 | 16.97 |
| ble004_merged | 13.55 | 5 | 536 | 620 | 10.85 | 16.24 |
| ble007 | 5.50 | 5 | 103 | 109 | 1.22 | 9.79 |
| ble008 | 12.96 | 5 | 188 | 216 | 8.48 | 17.44 |
| ble008_new_method | 10.0 | 5 | 99 | 110 | 4.39 | 15.61 |
| ble008_merged | 11.96 | 5 | 287 | 326 | 8.44 | 15.49 |

**Supplementary Table 5.** Mitochondrial contamination estimates after filtering of BLE samples

| Sample | Point est | Informative | Consensus | total | Lower CI | Higher CI |
| --- | --- | --- | --- | --- | --- | --- |
|  | (%) | sites | sites | alleles |  |  |
| ble004_nondr | 6.8 | 5 | 151 | 162 | 2.92 | 10.66 |
| ble004_dr | 0.77 | 4 | 257 | 259 | 0.0 | 1.84 |
| ble004_merged | 3.08 | 5 | 409 | 422 | 1.43 | 4.73 |
| ble007 | 1.23 | 4 | 80 | 81 | 0.0 | 3.63 |
| ble008 | 8.67 | 5 | 158 | 173 | 4.48 | 12.87 |
| ble008_new_method | 6.9 | 4 | 81 | 87 | 1.57 | 12.22 |
| ble008_merged | 8.61 | 5 | 244 | 267 | 5.25 | 11.98 |

**Supplementary Table 6**. READ results

| Individual pairs | Relationship | Z_upper | Z_lower |
| --- | --- | --- | --- |
| ble004-ble007 | Second Degree | 0.400436155785 | -1.44240939939 |
| ble004-ble008 | Unrelated | NA | -1.29951098643 |
| ble007-ble008 | Unrelated | NA | -3.02599405576 |

**Supplementary Table 7**. Number of SNP’s that overlaps with Human Origins SNP panel

| Individual | Population group | Abbreviation in this article | Assession numbers | Number of SNP | Reference |
| --- | --- | --- | --- | --- | --- |
| Huseby Klev ble004 | BLE | ble004 | PRJEB30667 | 20103 | This study |
| Huseby Klev ble007 | BLE | ble007 | PRJEB30667 | 19665 | This study |
| Huseby Klev ble008 | BLE | ble008 | PRJEB30667 | 52211 | This study |
| Yuzhnyy Oleni Ostrov, Karelia | EHG | I0061 | I0061/UzOO74 | 103493 | Mathieson et al. (2015) |
| Lebyanzhinka IV, Sok River, Samara | EHG | I0124 | I0124/SVP44 | 58629 | Mathieson et al. (2015) |
| Yuzhnyy Oleni Ostrov, Karelia | EHG | I0211 | I0211/UZOO40 | 20557 | Mathieson et al. (2015) |
| Hummervikholm en Hum1 | SHG | H22 | ERS1836020 | 45911 | **Günther** et al. (2018) |
| Hummervikholm en Hum2 | SHG | H26 | ERS1836021 | 106303 | **Günther** et al. (2018) |
| Steigen | SHG | Stg001 | ERS1836026 | 85224 | **Günther** et al. (2018) |
| Kanaljorden Motala12 | SHG | Motala12 | I0017 | 97330 | Lazaridis et al. (2014) |
| Kanaljoden Motala1 | SHG | Motala1 | I0011 | 69115 | Lazaridis et al. (2014) |

| Kanaljorden Motala2 | SHG | Motala2 | I0012 | 84900 | Lazaridis et al. (2014) |
| --- | --- | --- | --- | --- | --- |
| Kanaljorden Motala3 | SHG | Motala3 | I0013 | 53337 | Lazaridis et al. (2014) |
| Kanaljorden Motala4 | SHG | Motala4 | I0014 | 84852 | Lazaridis et al. (2014) |
| Kanaljorden Motala6 | SHG | Motala6 | I0015 | 73340 | Lazaridis et al. (2014) |
| Stora Bjers | SHG | Sbj001 / SBj | ERS1836022 | 39341 | **Günther** et al. (2018) |
| Stora Förvar Sf913 | SHG | Sf913 / SF9 | ERS1836025 | 83814 | **Günther** et al. (2018) |
| Stora Förvar Sf11 | SHG | Sf11 / SF11 | ERS1836023 | 19841 | **Günther** et al. (2018) |
| Stora Förvar Sf12 | SHG | SF12 / SF12 | ERS1836024 | 110573 | **Günther** et al. (2018) |
| Loschbour | WHG | Loschbour | ERS525703 | 110729 | Lazaridis et al. (2014) |
| LaBrana | WHG | LaBrana | SRS907006 | 105870 | Olalde et al. (2014) |
| Bichon | WHG | Bichon | ERS916239 | 110621 | Jones et al. (2015) |

### Supplementary Note 1

Archaeological background

According to recent research, there is substantial archaeological evidence of a dual-route colonization of the Scandinavian Peninsula at the end of the last Ice Age: one initial migration from the south c. 11,500 calBP, and a second migration from the northeast c. 10,300 calBP ^1-5^. The two routes are associated distinct technologies for producing lithic blades. In Scandinavia, the Early Mesolithic (c. 11,500-10,300 calBP) lithic blade technology traditionally associated with the Fosna and Hensbacka find groups, is characterized by blade detachment from one-sided, single and dual platform cores by direct percussion techniques.

The lithic technology and tool inventory show affiliation with the Final Palaeolithic Ahrensburgian tradition of northwestern Europe, suggesting a southern origin for the first settlers ^6-11,3,12^.

The later northeastern route is associated with the spread of pressure blade technology into Scandinavia, a technology known in preceding centuries from the East European Plain, Karelia, and Finland. This technology meant lithic blade production from conical and sub-conical cores with faceted platforms by means of pressure and indirect percussion techniques ^1-3^. The pressure blade technology appears to have been part of a technological package, that also included, for example, new concepts for crafting composite bone tools ^13-^ ^15^. Birch bark pitch was used as a glue in the composite bone tool technology ^16-17^.

Although changes in the lithic inventory at the transition from Early Mesolithic (c. 11,500-10,300 calBP) to Middle Mesolithic (c. 10,300-8300 calBP, known as Sandarna period in West Swedish chronology) have been recognized within Scandinavian Mesolithic research for long, these changes have traditionally been characterized as gradual internal developments ^18-19^. During recent years, the pressure blade technology has been documented for a large number of sites dated to c. 10,300 calBP and onwards in northern and western Scandinavia ^1,^ ^20-21,^ ^2-3,^ ^5^. The introduction of the pressure blade technology is suggested to represent the first migration of people and/or transmission of technological knowledge into Scandinavia from northwestern Russia and eastern Fennoscandia ^1-5^. The leading hypothesis is that the technology was first spread into northern Fennoscandia from northwestern Russia by migrating groups, and then dispersed rapidly southwards along the Norwegian coast into central and western Scandinavia with migrating groups and/or as knowledge transmitted from the immigrants to local population ^2^. This hypothesis is supported by a recent aDNA study ^22^, which shows that Mesolithic Scandinavian hunter-gatherers (SHG) have genetic traces from both eastern (EHG) and western (WHG) groups in different proportion. A direct link between the genetics of the ancient individuals and the lithic technology remains, however, to be established. None of the individuals studied for aDNA is directly associated with eastern blade production technology.

At the moment, the only direct link between genetics and Mesolithic stone tool technology was in the Stora Förvar cave, dated to 9460-8000 calBP (see Supplementary Note 2 for radiocarbon dates), on Gotland ^22^ and the Kanaljorden site in Motala, dated to 7840-7500 calBP, in eastern central Sweden ^23-26^. The other studied SHG individuals either have no association with lithic artefacts (Steigen, Hummervikholmen) or the associated artefacts are not sufficient to make inferences about stone tool production technology known to the buried individual (Stora Bjers)^22^.

The lithic material from Stora Förvar does, however, display affinity with the Maglemosian technogroup I and II ^27^ in Southern Scandinavia ^28,22^, characterized by a lithic technology similar to the technology used by the first Postglacial pioneers entering the Scandinavian Peninsula from the south.

Occasional examples of slotted bone points and blades possibly produced with pressure technique, were recovered from the Stora Förvar cave and Stora Bjers burial as single objects, comprising of two slotted bone points from the Mesolithic layers of Stora Förvar and a fragmented slotted bone point with a regular microblade from Stora Bjers ^28^.

However, no diagnostic production waste related to the eastern pressure blade technology was found. In Kanaljorden the lithic inventory has characteristics typical of the chronologically later handle core technology ^29^, a blade production technology typical of Late Mesolithic Scandinavia. Furthermore, the above mentioned finds are from a considerably younger period (c. 9400-7500 calBP) ^28^, leaving a gap of roughly 1000 years or more between these finds and the earliest documented finds of pressure blade technology in northern and western Scandinavia.

The Huseby Klev site

The Mesolithic Huseby Klev site was excavated in 1992-1994, prior to roadbuilding on the Orust island located in Bohuslän on the west coast of Sweden (Supplementary Figure 8, Supplementary Figure 9 and Supplementary figure 10). The excavation was never properly published. There is one short preliminary report in English ^30^, one summary report in Swedish ^31^ and one popular popular science book in Swedish ^32^. The excavation was led by Bengt Nordqvist from the Swedish National Heritage board. "Klev" is a local word for a small side valley, in this case a side valley for the larger Morlanda valley.

The excavated area was roughly 20,000 square meters (Supplementary Figure 16 a). One of the studied sectors, representing the site’s earliest dated excavated context, is a trench called the "deep pit", dated to c. 10,200-9400 calBP. In all, 1849 flint artefacts, 115 mastic pieces, and a large material of organic finds was retrieved from the deep pit. The site is assigned to the Sandarna period in the West Swedish chronological framework ^19^.

## The dating of the site

The finds from the Huseby Klev deep pit trench were found at c. 23 meters above sea level (masl) ^31^. This means that the findspot was submerged during the c. 10,200-9400 calBP use-span indicated by the radiocarbon dates from the site. That the location was submerged at the time is also confirmed by diatom studies of the find layer, which indicates the presence of salt-water sediments ^33^. The most likely scenario is that the sea retreated down to c. 27,5 masl, and at that time there was a shorebound site above the find location. The finds from the deep pit are consequently in part the result of waste thrown out into the sea, while some of the finds could also result from erosion that took place when the sea started to rise at c. 9600 calBP. This may be the case with the human bones, which are likely to be the remains of eroded graves. After c. 9600 calBP the sea level consistently rose up to 34,5 masl, during which the find layer of the deep pit was covered with one meter of clay sediments.

During the Neolithic a shorebound site was located at 25 masl on top of the sediments in the deep pit. There are 34 radiocarbon dates from the Huseby Klev excavation, six are from features post-dating the Mesolithic occupation, and are not discussed here.

The earliest date, c. 10,800-10,600 calBP (Ua-5654) is on a piece of charcoal from a transgressed layer in the northern part of the site (89N). Twenty-two square meters of this layer were excavated, while only a relatively small amount of finds were recovered (c. 100 flints). The exact height above sea level of this find spot is not known.

Five of the 28 Mesolithic radiocarbon dates are made on human (4) or whale (1) bones. These are among the earliest dates from the site but this is, at least partly, a consequence of the reservoir effect, since stable carbon and nitrogen isotope analyses on the human bones show that the diet consisted mainly of marine resources (Supplementary figure 15) ^34^. For whales in the NW Atlantic Ocean the reservoir effect is, nowadays, c. 350+/-50 years ^35^. We follow Günther et al. ^22^ and use a reservoir correction of 380±30 years for the human and whale bones from Huseby Klev (Supplementary Note 2, Supplementary Excel Table 4). From the transgressed layer in the deep pit there are eight radiocarbon dates on hazelnut shell and two on birch bark pitch (mastic) pieces, both are terrestrial materials and give reliable dates. The span of

radiocarbon date median values is wide; c. 10,040 - 9610 calBP (Supplementary Note 2, Supplementary Excel Table 4) and corresponds with a period with low sea level in the shoreline curves from Ljungskile and Göteborg ^36-37^. It is probable that the finds derive from a shore bound site used during this period. During the following transgression the layers were sealed at c. 9600 calBP. It is therefore clear that all the aDNA-analysed mastic pieces in this study predate the transgression. This assumption is confirmed by two mastic pieces from the layer, with the combined date 9880-9540 calBP (Ua-56731 and Ua-7156 combined with the r-combine function in OxCal 4.3, see Supplementary Excel Table 4 for separate dates).

There are no shore displacement studies conducted at or close to the Huseby Klev site. The geographically closest shore displacement studies are mapped in Supplementary Figure 11. None of the available curves can be used directly for dating the Huseby Klev site, but they give a geological background and give strength to the chronological frame indicated by the radiocarbon dates.

In order to utilize an established shoreline curve in areas other than the one it is constructed for, the direction of lines connecting areas with comparable shoreline displacement must be considered (isobars). There are two sets of data that are commonly used to determine the isobars; the present rate of land upheaval and highest shorelines. Miller and Robertsson ^40^ have compiled the available isobar data for the area close to Huseby Klev. In North Bohuslän Påsse ^40^ used a line perpendicular to N30degreeE. If compared with the Miller and Robertsson ^38^ study, the N30degreeE line seems applicable in the coastal area, but not further inland. This means that among the sites with shoreline displacement curves in Supplementary Figure 11, the Risveden curve cannot be used to make conclusions about shoreline displacement at Huseby Klev.

The Central Bohuslän data presented by Miller and Robertsson ^38^, is not very detailed for the period c. 11,000-8000 calBP . It shows a more or less constant shore level at c. 40 meters above present sea level for this period. The Ljungskile study covers the period c. 11,000-8000 calBP. Ljungskile is located on an isobar 18.3 km further north (measured N30degreeE, see Supplementary Figure 11), and therefore the shore level during the studied period can be expected to have been lower at Huseby Klev. The Ljungskile diagram (actually for lake Kolbengtseröd) by Persson ^36^, shows a series of minor peaks and drops in the shore level between c. 10,000 and 8000 calBP ending in a transgression (i.e., a period when the sea level rise is faster than the postglacial land upheaval, causing inundation of earlier shorelines). At Ljungskile this transgression goes up to 42 masl. Further south in Göteborg the transgression reached 23 masl ^37^. The difference in height is 19 m. Huseby Klev is located at 3/5 of the distance between Göteborg and Ljungskile. Extrapolation therefore indicates transgression up to c. 34,4 masl at Huseby Klev. This can be tested against two of the observations made by Persson ^36^; lake Hogen with a transgression maximum at 39 masl and lake Tjärnevattnet with a maximum at 30 masl. Huseby Klev is located at an equal distance from both ends on a straight line drawn between the two lakes, giving 34,5 masl as the transgression maximum at Huseby Klev. The extrapolation between Ljungskile and Göteborg indicates that the lowest lying shoreline before the transgression at Huseby Klev would have been at c. 27,6 meters above present sea level. The transgression maximum is dated to c. 8200 calBP and the lowest level during the preceding regression to c. 10,300 calBP (the dates in Påsse ^37^,

calibrated). At Huseby Klev the shore retreated after the transgression, and at 25 masl there is a cultural layer with Neolithic finds radiocarbon dated to c. 6000 calBP (Ua-5658). Taken together, the above described reasoning can be summarized in the approximate shoreline displacement curve for Huseby Klev illustrated in Supplementary Figure 12.

## Lithic technology

In all, 1849 pieces of worked flint were collected from the transgressed layers in the deep pit of Huseby Klev during excavation. In order to investigate the link between the genetics of the ancient individuals and

the lithic technology used at the site, technological analysis of the lithic assemblage was conducted (Supplementary table 9). A central question was if the lithic assemblage from the deep pit is affiliated with the eastern pressure blade technology. In order to resolve this, the lithic blade production concept was reconstructed by defining the production methods and knapping techniques used at the site.

Blade production methods and techniques are deduced from the archaeological record through very different procedures. While recognition of the production method depends on technological reading of *debitage* products and positioning each artefact in the operational chain (chaîne opératorie) of production, the identification of knapping techniques is based on specific blade and core attributes found by experimental work and by analogy recognised in prehistoric lithic assemblages ^41-51,3^ . Central blade and core attributes discussed in the text are shown in Supplementary Figure 13.

## Method and sampling

The technological analysis includes 1) a dynamic-technological classification by which the lithic artefacts are classified according to their stage in the production process, and 2) a classification of specific technical attributes through a simplified *chaîne opératoire* analysis of the complete lithic assemblage from the site, as well as 3) an attribute classification of a selection of the artefacts ^52-53,27,49,54,51,3,12^. The attribute classification follows the Nordic Blade Technology Network (NBTN) standard reference sheet *Dynamic technological Classification of Scandinavian Lithic Blade Industries- second edition* (Mikkel Sørensen, NBTN, March 2013), with certain modifications ^3^. Altogether 86 artefacts that, according to a dynamic technological understanding of how blades can be produced and the sequential reduction of lithic materials, were considered high-priority in determining the blade production methods and knapping techniques used, were subsequently selected and catalogued according to the attribute classification. The catalogued artefacts include 57 blades and proximal blade fragments, 13 blade cores, four core fragments, eight platform rejuvenation flakes and four formal blade tools.

## Operational chain of production

The different stages of an operational chain of lithic tool production can be summarized as involving four main stages: raw material procurement, production, utilization/use and discard ^55,53^. Strategies for raw material use and procurement represent the first stage in the operational chain, and form the basis of the production process. Production is the second stage and comprises both primary and secondary core preparation and exploitation, as well as the production of blade blanks and tools. These two initial stages form the foundation on which the analysis is consequently built.

The catalogued artefacts were, initially, sorted according to flint types and a minimum of six different types were used for blade production, including Maastrichtian flint, as well as beach-flint nodules of varying qualities that were probably collected nearby. The raw material consists mainly of high workability flint. In general, all stages of the blade production process are represented at the site; waste from the primary and secondary preparation of cores, all stages of blade production, and discarded blade blanks and tools.

The lithic assemblage consists, however, mainly of artefacts related to the primary and secondary stages of core preparation and exploitation. Testing and discarding of raw material in its more or less original unworked condition, is present in the form of unworked and tested beach-flint nodules, as well as core preforms. The lithic assemblage is characterized by a high frequency of cortical blades and flakes, crested blades, core preparation waste related to primary core exploitation, as well as cores and blades from the initial and early stages of production. Artefacts related to the secondary and final stages of blade blank and blade tool production are present at a relatively low frequency, suggesting that prepared cores were primarily transported away from the site. Some blade production and re-tooling was, however, performed on-site, visible by the presence (in relatively low numbers) of discarded tools and regular blade blanks.

## Blade production method

The core assemblage consists mostly of precores and cores from the early stages of production. Blade production involved production from single-platform conical and sub-conical cores with elongated fronts. 13 sub-conical blade cores, fragments of one conical core and three sub-conical cores, as well as eight platform rejuvenation flakes were catalogued. Complete cores from the final stage of regular blade production were not observed in the studied assemblage. The complete cores measure between 2.4-6.0 cm in height and 2.0-4.4 cm in width.

Large cortical flakes and blades, including crested blades with areas of remaining cortex, indicate that the preparation of cores involved removal of cortical blades, and unifacial and bifacial removals from a crested ridge along the lateral side of the core. Cresting appears to have been a common strategy for preparing cores and retaining core geometry throughout the production sequence. The cores have both wide (n=10) and narrow (n=3) fronts, with mostly ¾ (n=8) and one-sided (n=4) front exploitation. One core has circular front exploitation but, due to the way the platform was prepared, it appears to have been exploited from one side during each sequence. The core fronts were rejuvenated by removing large, broad blade-like flakes, as well as the removal of distal rejuvenation flakes for corrections of the lower parts of the front. The shape and character of the core back varies. The backsides are curved or flat, partly or completely flaked in order to flatten the side opposite to the core face.

Core platforms are primary concave (n=7) or flat (n=4). The platform to front angle varies, with a 84° average. 73% of all cores and platform rejuvenation flakes and 44% of blades have a platform to front angle between 85-95°. The strategy for preparing the core platform prior to blade detachment consisted of both edge trimming and abrasion in order to rub down the overhang, a procedure visible in 82% of blades, as well as faceting the platform surface (in 69% of the cores). The majority of the platforms have large facets (n=8) and in two of the cases the preparation was restricted to the edge of the platform surface. In four cases the platforms were left unprepared. Only one core has preparation in the form of systematic faceting by the removal of small flakes that terminated in hinges towards the centre of the platform surface, a key feature of the eastern pressure blade technology ^2^ (Sørensen et al. 2013). This specific strategy is, however, represented at the site in the form of platform rejuvenation flakes and core fragments with systematic faceting. Six out of eight platform rejuvenation flakes have systematic faceting. Furthermore, all of the platform rejuvenation flakes have a circular shape and platform to front angle close to 90°, indicating that they derive from conical cores, and that blade production was either by indirect percussion or by pressure techniques. The strategy of small flake faceting of the platform surface is, however, not reflected in the waste material by large frequencies of small thin preparation flakes with hinged termination, a feature typical of the eastern pressure blade technology concept ^1-2^. Altogether 57 complete blades and proximal blade fragments were catalogued. Blades are in most cases straight (62%) with ideal termination. Blades with distal (18%) and even (18%) curvature are present, but in low numbers. 40% have proximal twisting indicating that the cores were supported distally during production. The frequency of straight (77%) and oblique (23%) dorsal profiles indicate that blades primarily were removed from the core front, but also that the sides were exploited. During the technological analysis only a selection of the blades were catalogued, but according to visual examination, complete blades and proximal fragments dominate. The frequency of distal and medial fragments is relatively low. All blades were measured for their length, width and thickness. The distribution of blade length and width indicates a generalised strategy involving a gradual reduction of the core to produce blanks of different sizes. The complete blades measure 2.7- 8.5 cm in length, while the average is 5.2 cm. Blade width varies between 0.7-2.9 cm, and there is no indication of separate macro- and microblade production. The average blade width is 1.4 cm and the average thickness is

0.5 cm.

## Knapping techniques

Blades are in general regular (52%) to irregular (30%), whereas the remaining 18% are extremely regular. The ventral platform angle varies with an average of 81°, but 44% have a ventral angle close to 90°. Blades commonly have diffuse lips (68%) and diffuse bulbs (50%), and a low frequency of bulbar scars (25%). Bulbs are both long and diffuse, or high and short, often with a marked ripple under the bulb. However, 43% have conus formation, primarily in the form of ventral fissures (37%). Butt morphology varies, but thin oval butts (47%) dominate. The majority of the blades display characteristic in accordance with blades produced with direct and indirect percussion techniques ^56^. Cortex and crested blades from initial blade production display diagnostics typical for blades produced by direct percussion technique, being broad, thick, and irregular with pronounced bulbs. Also regular, straight to slightly curved blades with diagnostic features of indirect technique are present. A relatively low percentage of the blades has diagnostic features indicating production by pressure technique. Such blades being straight, regular to very regular, thin with diffuse lip and with high, short and pronounced bulb, a thin oval butt, and a ventral platform angle of 90°.

## Blade production concept

The blade production concept documented for the deep pit of Huseby Klev displays clear affiliation with the eastern pressure blade technology, as documented for a large number of sites in northern and western Scandinavia, eastern Fennoscandia, and the East European Plain ^1,20-21,2-4^. In general, the blades appear to have been produced by the same overall concept: serial production from single-platform, sub-conical and conical cores with both faceted and smooth platforms, by means of indirect and pressure technique in combination with direct percussion techniques. The variation in knapping techniques was related to different stages in the production process. Direct and indirect percussion techniques were used for initial core preparation and in the early stages of blade production as well as for corrections. Pressure technique was used in the middle and final stages of the production sequence. Morphometric analysis shows the production of a consistent range of blade blanks, which in turn allowed the production of standardized tools such as barbed points (hulling-type) and slender lanceolate microliths, as well as blades with lateral retouch on one or both edges. The last mentioned were probably used as inserts in composite slotted tools, to which the inserts were attached using mastic made of birch bark pitch ^57-58^.

The blade production concept represented by the Huseby Klev deep pit finds appears, however, somewhat less distinct than the pressure blade technology in many other sites in northern and western Scandinavia. Features considered diagnostic for the eastern pressure blade technology, are relatively low in the deep pit. These include the frequencies of core rejuvenation flakes, the small hinged preparation flakes from faceting the platform surface, and the short medial blade fragments with perpendicular breaks from intentional snapping of blades into rectangular segments. The proportion of very regular thin blades produced with pressure technique, as well as heavily exploited blade cores are also small. Nevertheless, all the diagnostic features of the eastern pressure blade concept are present in the material (Supplementary Figure 14), suggesting that the explanation for the variation can most probably be found in functional differences or site formation processes. No artefacts diagnostic to the Early Mesolithic blade production concept, that would indicate that the lithic assemblage is chronologically or technologically mixed, were observed. Rather, the character of the artefacts suggests that they derive from the same technological concept. The lithic artefacts from the deep pit are partly considered to be the result of waste disposal in the sea from the settlement area. This may have resulted in sorting of the material, meaning that the bulk of smaller pieces, such as preparation flakes and medial blade fragments, are located outside the excavated area and were thus not collected during the excavation.

The aforementioned dissimilarities in lithic material composition between the deep pit of Huseby Klev and the majority of the other investigated sites where the pressure blade technology was in use, are most likely the result of site formation processes along with site-specific behavior, because of which the different stages of the operational chain of blade production were performed at different sites. The artefacts from the deep pit appear primarily to originate from the primary and secondary stages of core preparation and exploitation. Artefacts related to the secondary and final stages of blade blank and blade tool production are represented in relatively low frequencies. Based on the composition of lithic artefact types, the site appears to represent a production site into which lithic raw material in its more or less unworked condition was transported and initial core preparation performed. Prepared cores (and blade blanks) appear to have been mostly transported away from the site. This may explain the relatively low numbers of blades produced by pressure technique, as well as the lack of thoroughly exploited blade cores and discarded blade tools. Some tool manufacture and re-tooling was, however, performed on site, indicated by a (low) number of discarded tools and regular blade blanks.

## Organic remains

In addition to the lithic artefacts, the deep pit yielded a rich collection of organic finds. The bone material from the deep pit is the earliest recovered from a sea shore context in Scandinavia ^59^. This is due to the marked sea level changes that took place after the latest Ice Age, which submerged all such sites in southern Scandinavia. The bone and antler tool inventory includes an axe, a pickaxe made of red deer antler, a bone arrowhead and two bone fish hooks. In addition, a wild boar tooth with a hole drilled in the root (probably a pendant), and a worked dolphin vertebra with a hole in the centre and possible traces of decoration, were found. Among the bone finds, 712 fragments have been identified to the genus or species level in a recent study by Boethius ^60^. Among hoofed mammals (130 fragments), the most common types were wild boar and red deer. Among marine mammal (206 fragments), the most common types were white-beaked dolphin, harbour porpoise and grey seal. Fish (268 identified fragments), were dominated by cod. The bird bones were mainly from auks, such as common murre and the flightless great auk. There were also dog bones and seven species that were hunted mainly for their fur.

In addition to the human bones that had been sorted out earlier, Boethius found two human bones among the animal bones. Stable isotope analysis has been conducted on the human bones ^34,^ ^61^. Five human bones and 22 animal bones from the deep pit have been measured for carbon-13 (C13) and nitrogen-15 (N15), Figure S1:8. The human bones group together with marine species. Boethius and Ahlström ^61^ have argued that this should be interpreted as a diet consisting of 51-55% marine high trophic fish (like cod) and 24- 28% marine mammals, while the rest (i.e. <25%) of the diet would consist of terrestrial animals and plants. The human bones from the deep pit have been screened for aDNA. A single bone showed good preservation and will be analysed further in a forthcoming study. Besides the bones, there were finds of edible plants: hazel, apple, blackthorn, rose hip, and bird cherry. Worked pieces of wood and wooden sticks with one burnt end, possibly used as torches for fishing, were also recovered.

## The mastics

One artefact group of special interest here is the set of 115 mastic pieces found in the deep pit, of which 84 were described in detail ^31^. The distribution of all mastic pieces found during excavation is shown in Supplementary Figure 16. Besides the mapped pieces, a number of mastics were found in soil samples collected after excavation and therefore not included in the excavation report. Three of these pieces were used in this study.

Technologies that employ more or less processed cohesive substances as an adhesive mastic, are known from around the world from the Middle Palaeolithic onwards ^62-71^. According to analyses conducted on the

chemical composition of prehistoric adhesives, in most cases pitch produced from birch bark (Betula sp.) was used in Europe ^66-67,72-73,58^, while teeth impressions found on a number of lumps of birch bark pitch show that they were masticated in prehistoric times ^74,67,75^. Of the more than 115 finds of pitch from Huseby Klev, eight lumps from the deep pit have been subjected to chemical analysis ^57^. Seven of them turned out to be birch bark pitch while one did not give results. Alexandersen ^76^ has studied 10 lumps with tooth impressions and determined the age of the chewers in these cases to have been between 5-18 years by comparing to tooth development and wear to modern parallels. In addition, a piece with teeth marks from both an adult and a child has been reported ^32^. Other pieces of pitch from the deep pit show different kinds of impressions from wood and cordage ^77^.

Ethnographic evidence of the chewing of different kinds of cohesive substances for medical or purely non- utilitarian purposes, is found in most parts of the world. Widely known are the consumption of *chicle* (i.e., the resin of the sapodilla tree, *Manilkara zapota)* and natural bitumen in the Americas ^78-79^, as well as the *Chios mastic, i.e.*, the aromatic resin of *Pistacia lentiscus* in the Mediterranean region ^80^. Less known is the chewing of resins of spruce (*Picea abies*) and sometimes pine (*Pinus sylvestris*) in Scandinavia and Finland

81-83 or the masticates produced from spruce, birch and larch (Larix sp.) among many Siberian peoples ^84,^ ^83,^

74. While modern experiments have shown that relatively simple methods can be used in the production of birch bark pitch ^85-89^, the production technology is somewhat knowledge-intensive. It is mostly for this reason that teeth marks in the pitch are often considered as indicative of processing and use, i.e., making the pitch more viscous and pliable, rather than a sign of purely recreational use as a "chewing gum" ^67^.

### Supplementary Note 2

Radiocarbon dates from SHG contexts

Altogether 20 individuals have been ascribed to the genetically defined Scandinavian hunter-gatherers group (SHG). Besides Huseby Klev, they derive from five locations and are dated between 9660-5860 calBP. All radiocarbon dates from the contexts that have yielded aDNA that groups with SHG are given in Supplementary Excel Table 4 (Dryad depository). We formed “context dates”, i.e., chronological ranges for site use, using median values of the earliest and latest radiocarbon date per context. Where possible, only dates on terrestrial material are used for the context date. For the Huseby Klev deep pit this span is 10,040- 9610 calBP. Since stable carbon and nitrogen isotope analyses on human bones from coastal Stone Age sites in the area show that the diet consisted mainly of marine resources ^34,^ ^90^. We follow Günther et al. ^22^, and Eriksson et al ^90^. and use a reservoir ^91^ correction of 380±30 years for human bones and bones of aquatic animals from Hummervikholmen, Huseby Klev, and Steigen, a 70±40 years correction for Stora Förvar and Stora Bjers, and no correction for Motala Kanaljorden.

*Kanaljorden, Motala (southern Sweden)*

The Kanaljorden site has yielded remains of 11 individuals in total ^91^. There are altogether 36 published radiocarbon dates from the site (Supplementary Excel Table 4), of which 19 are on human bones. A context date based on 15 dates on terrestrial animal bone give a span of 7760-7520 calBP, suggesting that there is no major reservoir effect in the dates made on human bones from Kanaljorden, which range between 7840- 7540 calBP, while most are between 7800 and 7600 calBP.

*Hummervikholmen (SE Norway)*

The Hummervikholmen site is situated c. 1 m below the present sea level ^92,^ ^93^. The finds consist of less than 50 pieces of human bones, from at least three individuals. There are altogether 32 published radiocarbon dates from the site, of which 11 are made on human bone, while the others have no secure

connection to human activity. There are no associated artefacts from the location. The human bones from Hummervikholmen have yielded radiocarbon dates ranging between 9540-8610 calBP, while most dates fall between 9360-9250 calBP (Supplementary Excel Table 4).

*Stora Förvar (Gotland, Sweden)*

The Stora Förvar cave site has yielded human bones from an unknown number of individuals. The early SHG individuals derive from a Mesolithic settlement layer in the cave. Altogether 47 radiocarbon dates have been published from the Mesolithic layer, of which 21 are on human bones, 17 are on aquatic animals, and 9 are on hazelnut shells and hare bones (Supplementary Excel Table 4). A context date based on based on the 9 terrestrial dates gives a span of 9170- 8000 calBP, while most dates are between 9040-8850 calBP. The dates made on human bones from Stora Förvar, which range between 9490-7310 calBP, while most dates fall between 9310-8920 calBP are relatively well in line with the context date.

*Stora Bjers (Gotland, Sweden)*

The Stora Bjers individual derives from a grave together with an antler axe ^94^. Altogether three dates have been published (Supplementary Excel Table 4). Two dates made on the skeleton both give median values of c. 8770 calBP, while a date made on the axe of red deer antler is 8590-8410 calBP. The difference between the dates suggests a greater than anticipated reservoir effect in the dates on human bone at this site.

*Steigen (northern Norway)* The SHG individual from Steigen is represented by a single human mandible found in a cave. Archaeological excavation at the find spot produced only a tooth fitting into the mandible and no associated artefacts. The mandible has been radiocarbon dated to 5960-5760 calBP (Supplementary Excel Table 4).

### References for Supplementary figures and tables

1. Rankama, T. & Kankaanpää, J. First evidence of eastern Preboreal pioneers in Arctic Finland and Norway. *Quartär* **58**, 183–209 (2011).
2. Sørensen, M. et al. The first eastern migrations of people and knowledge into Scandinavia: Evidence from studies of Mesolithic technology, 9–8 millennium BC. *Norwegian*

*Archaeology Review* **46**(1), 19–56 (2013).

1. Damlien, H. Between Tradition and Adaption. Long-term trajectories of lithic tool-making in South Norway during the postglacial colonization and its aftermath (c. 9500-7500 cal. BC). (Thesis. University of Stavanger, Stavanger, 2016).
2. Damlien, H. Eastern pioneers in westernmost territories? Current perspectives on

Mesolithic hunter-gatherer large-scale interaction and migration within Northern Eurasia.

*Quaternary International* **419**, 5–16 (2016).

1. Damlien, H., Kjällquist, M. & Knutsson, K. in *The Technology of Early Settlement in Northern Europe – Transmission of Knowledge and Culture. Vol. 2* (eds Knutsson, K., Knutsson, H., Apel, J. & Glørstad, H.) 99-137. (Equinox Publishing, Sheffield, 2018).
2. Kindgren, H. in *The Earliest Settlement of Scandinavia and its relationship with neighbouring areas* (ed Larsson, L.) 191-205 (Almquist & Wiksell International, Stockholm, 1996).
3. Schmitt, L. Comparative points and relative thoughts: The relationship between the Ahrensburgian and Hensbacka assemblages. *Oxford Journal of Archaeology* **18**(4), 327-337

(1999).

1. Bang-Andersen, S. Southwest Norway at the Pleistocene/Holocene Transition: Landscape Development, Colonization, Site Types, Settlement Patterns. *Norwegian Archaeological Review* **36**(1), 5-25 (2003).
2. Bjerck, H. B. in *Mesolithic Europe (*eds Bailey, G. & Spikins, P.) 60-106 (Cambridge University Press, Singapore, 2008).
3. Schmitt, L. et al. Chronological insights, cultural change, and resource exploitation on the West Coast of Sweden during the Late Palaeolithic/ Early Mesolithic Transition. *Oxford Journal of Archaeology* **28**(1), 1–27 (2009).
4. Fuglestvedt, I. The Pioneer condition on the Scandinavian Peninsula: the Last Frontier of “Palaeolithic Way” in Europe. *Norwegian Archaeological Review* **45**(1), 1-29 (2012).
5. Berg-Hansen, I. M. in *The Technology of Early Settlement in Northern Europe – Transmission of Knowledge and Culture. Vol. 2* (eds Knutsson, K., Knutsson, H., Apel, J. & Glørstad, H.) 63–98 (Equinox Publishing, Sheffield, 2018).
6. Bergsvik, K. A. & David, E. Crafting Bone Tools in Mesolithic Norway: A Regional Eastern- Related Know-How. *European Journal of Archaeology* **18**(2), 190-221 (2016).
7. Knutsson, H., Knutsson K., Molin, F. & Zetterlund, P. From flint to quartz: Organization of lithic technology in relation to raw material availability during the pioneer process of Scandinavia. *Quaternary International* **424**, 32-57 (2016).
8. David, E. & Kjällquist, M. in *The Technology of Early Settlement in Northern Europe – Transmission of Knowledge and Culture. Vol. 2* (eds Knutsson, K., Knutsson, H., Apel, J. & Glørstad, H.) 231-276. (Equinox Publishing, Sheffield, 2018).
9. Edgren, T. in *Till Gunborg. Arkeologiska samtal* (eds Åkerlund, A., Bergh, S., Nordbladh, J. & Taffinder, J.) 22-38 (Stockholm University, Stockholm, 1997).
10. Zhilin, M. Early Mesolithic Bone Arrowheads from the Volga-Oka interfluve, Central Russia. *Fennoscandia archaeologica* **XXXII**, 35-54 (2015).
11. Bjerck, H. B. The Fosna-Nøstvet problem: a consideration of archaeological units and chronozones in the South Norwegian Mesolithic Period*. Norwegian Archaeological Review* **19**(2), 103–121 (1986).
12. Nordqvist, B. *Coastal adaptions in the Mesolithic. A study of coastal sites with organic remains from the Boreal and Atlantic periods in Western-Sweden*. GOTARC. Series B. Gothenburg archaeological thesis. No 13. (Göteborg, 2000).
13. Rankama, T. & Kankaanpää, J. in *Early Economy and Settlement in Northern Europe.*

*Pioneering, Resource Use and Coping with Change, Vol. 3* (ed Blankholm, H.P.) 139-168 (Equinox Publishing, Sheffield, 2018).

1. Knutsson, H. & Knutsson, K. The Postglacial colonization of humans, fauna and plants in northern Sweden. *Arkeologi i Norr* **13** (Umeå, 2012).
2. Günther, T. et al. Population genomics of Mesolithic Scandinavia: Investigating early postglacial migration routes and high-latitude adaptation. *PLOS Biology* **16**(1), e2003703 (2018).
3. Lazaridis, I. et al. Ancient human genomes suggest three ancestral populations for present-day Europeans. *Nature* **513**(7518), 409–413 (2014).
4. Haak, W. et al. Massive migration from the steppe was a source for Indo-European languages in Europe. *Nature* **522**(7555), 207–211 (2015).
5. Mathieson, I. et al. Genome-wide patterns of selection in 230 ancient Eurasians. *Nature*

**528**(7583), 499–503 (2015).

1. Mathieson, I. et al. The genomic history of southeastern Europe. *Nature* **555**(7695), 197– 203 (2018).
2. Sørensen, M. in *Stenalderstudier. Tidligt mesolitiske jægere og samlere i Sydskandinavien* (ed Eriksen, B. V.) 19-76 (Jysk Arkæologisk Selskab, Højbjerg, 2006).
3. Apel, J. & Storå, J. in *The Ecology of Early Settlement in Northern Europe. Vol 1* (eds Persson, P., Riede, F., Skar, B., Breivik, H. M. & Jonsson, L.) 277-310 (Equinox Publishing, Sheffield 2018).
4. Knutsson, H., Knutsson, K. & Damlien, H. in Kanaljorden, Motala – Rituella våtmarksdepositioner och boplatslämningar från stenåldern (ed Hallgren, F.) (Stiftelsen Kulturmiljövård, Västerås, in press).
5. Nordqvist, B. in *News Warp. The Newsletter of the Westland Archaeology Research Projekt* 16 (ed Coles, J.) (1994) <http://newswarp.info/>
6. Nordqvist, B. *Huseby klev. En kustboplats med bevarat organiskt material från äldsta mesolitikum till järnålder. Bohuslän, Morlanda socken, Huseby 2:4 och 3:13, RAÄ 89 och 485*.

(Riksantikvarieämbetet. UV Väst, Mölndal, 2005).

1. Hernek, R. & Nordqvist, B. *Världens äldsta tuggummi? Ett urval spännande arkeologiska upptäckter som gjordes vid Huseby klev, och andra platser, inför Väg 178 över Orust* (Riksantikvarieämbetet. UV Väst, Kungsbacka, 1995).
2. Svedhage, K. in *Huseby klev. En kustboplats med bevarat organiskt material från äldsta mesolitikum till järnålder. Bohuslän, Morlanda socken, Huseby 2:4 och 3:13, RAÄ 89 och 485* (ed Nordqvist, B.) 109-112 (Riksantikvarieämbetet. UV Väst, Mölndal, 2005).
3. Lidén, K., Eriksson, G., Nordqvist, B., Götherström, A. & Bendixen, E. “The wet and the wild followed by the dry and the tame” - or did they occur at the same time? Diet in Mesolithic - Neolithic southern Sweden. *Antiquity* **78**(299), 23–33 (2004).
4. Mangerud, J., Bondevik, S., Gulliksen, S., Hufthammer, A.K. & Høisæter, T. Marine 14C reservoir ages for 19th century whales and molluscs from the North Atlantic. *Quaternary Science Reviews* **25**(23–24), 3228–3245 (2006).
5. Persson, G. *Postglacial transgressions in Bohuslän, Southwestern Sweden* (Vol. 684) (University of Gothenburg, Gothenburg, 1973).
6. Påsse, T. *Havsstrandens förändringar i norra Halland under Holocen tid* (Vol. A45). (University of Gothenburg, Gothenburg, 1983).
7. Miller, U. & Robertsson, A.-M. Late Weichselian and holocene environmental changes in Bohuslän, southwestern Sweden. *Geographia Polonica* **55**, 103–111 (1988).
8. Svedhage, K. *Shore displacement during the late Weichselian and early Holocene in the Risveden area, SW Sweden* (Vol. 51) (University of Gothenburg, Gothenburg, 1985).
9. Påsse, T. in *Strandlinjer och vegetationshistoria. Kvartärgeologiska undersökningar inom Kust till kust projektet, 1998-2002* (ed Persson, P.) 31–87 ( Arkeologiskt Naturvetenskapliga

Laboratoriet, Gothenburg, 2003).

1. Crabtree, D. E. Notes on Experiments in Flintknapping. *Tebiwa* **10**, 60–73 (1967).
2. Bordes, F. & Crabtree, D. E. The Corciac Blade Technique and Other Experiments. *Tebiwa*

**12**(2), 1–20 (1969).

1. Callahan, E. Experiments with Danish Mesolithic Microblade Technology. *Journal of Danish Archaeology* **4**, 22-39 (1985).
2. Madsen, B. Nogle nomenklatoriske bemærkninger til studiet af flintteknologi - eksperimentelt og arkæologisk. *Fjølnir* **5**(1), 3-28 (1986).
3. Migal, W. in *Skilled Production and Social Reproduction - Aspects on Traditional Stone Tool Technologies* (eds Apel, J. & Knutsson, K.) 387–398 (Societas Archaeologica Upsaliensis, Uppsala, 2006)
4. Pelegrin, J. in *Mesoamerican Lithic Technology: Experimentation and Interpretation* (ed Hirth, K.) 55–71 (University of Utah Press, Salt Lake City, 2003)
5. Pelegrin, J. in *Skilled Production and Social Reproduction - Aspects on Traditional Stone Tool Technologies* (eds Apel, J. & Knutsson, K.) 37–68 (Societas Archaeologica Upsaliensis, Uppsala, 2006).
6. Pelegrin, J. in *The Emergence of Pressure Blade Making. From Origin to Modern Experimentation* (ed Desrosiers, P. M.) 465-496 (Springer, New York, 2012)
7. Sørensen, M. in *Skilled Production and Social Reproduction - Aspects on Traditional Stone Tool Technologies (*eds Apel, J. & Knutsson**,** K.) 277–299 (Societas Archaeologica Upsaliensis, Uppsala, 2006).
8. Sørensen, M. *Technology and Tradition in the Eastern Arctic, 2500 BC-AD 1200. A Dynamic Technological Investigation of Lithic Assemblages from the Paleo-Eskimo Traditions*

*of Greenland* (University of Chicago Press, Chicago, 2012).

1. Eigeland, L*.* Maskinmennesket i Steinalderen. Endring og kontinuitet i steinteknologi fram mot neolitiseringen av Øst-Norge (Thesis. University of Oslo, Oslo, 2015).
2. Schild, R. in *Unconventional archaeology: new approaches and goals in Polish archaeology* (ed Schild, R.) 57–87 (Zakład Narodowy im. Ossolińskich, Wrocław, 1980).
3. Eriksen, B. V. in *Flintstudier. En håndbog i systematiske analyser af flintinventarer*. (ed Eriksen, B. V.) 75-101 (Aarhus Universitetsforlag, Aarhus, 2000).
4. Sørensen, M. Introduction to the Dynamical technological classification of Scandinavian lithic blade assemblages. Nordic Blade Technology Network (Unpublished report, 2013).
5. Inizan, M. L., Reduron-Ballinger, M., Roche, H. & Tixier, J. *Technology and terminology of knapped stone* (CREP, Nanterre, 1999).
6. Damlien, H. Striking a Difference? The Effect of Knapping Techniques on Blade Attributes. *Journal of Archaeological Science* **63**, 122-135 (2015).
7. Aveling, E. M. *Characterisation of Natural Products from the Mesolithic of Northern Europe. Chemical Analysis of Amorphous Organic Materials from a Number of Sites in Britain*

*and Scandinavia* (Thesis. University of Bradford, 1998)

1. Vahur, S., Kriiska, A. & Leito, I. Investigation of the adhesive residue on the flint insert and the adhesive lump found from the Pulli Early Mesolithic settlement site (Estonia) by

micro-ATR-FT-IR spectroscopy. *Estonian Journal of Archaeology* **15**(1), 3-17 (2011).

1. Mansrud, A. & Persson, P. in *The Ecology of Early Settlement in Northern Europe, Vol 1* (eds Persson, P., Riede, F., Skar, B., Breivik, H. M. & Jonsson, L.) 129–165 (Equinox Publishing, Sheffield, 2018).
2. Boethius, A. in *The Ecology of Early Settlement in Northern Europe, Vol. 1* (eds Persson, P., Riede, F., Skar, B., Breivik, H. M. & Jonsson, L.) 129–165 (Equinox Publishing, Sheffield, 2018).
3. Boethius, A. & Ahlström, T. Fish and resilience among Early Holocene foragers of

southern Scandinavia: A fusion of stable isotopes and zooarchaeology through Bayesian mixing modelling. *Journal of Archaeological Science* **93**, 196-210 (2018).

1. Webb, W.S. *Indian Knoll, Site OH 2, Ohio County, Kentucky. William S. Webb.* Reports in Archeology and Anthropology 4-1 (University of Kentucky, Lexington, KY, 1946).
2. Mason, O. T. et al. Arrows and Arrow-Makers. *American Anthropologist* **4**(1), 45-74 (1891).
3. Binford, L. R. An Alyawara Day: Flour, Spinifex Gum, and Shifting Perspectives. *Journal of Anthropological Research* **40**(1), 157-182 (1984).
4. Hayek, E. W. H. et al. Identification of archaeological and recent wood tar pitches using gas chromatography/mass spectrometry and pattern recognition. *Analytical Chemistry*

**62(**18), 2038–2043 (1990).

1. Aveling, E. & Heron, C. Identification of birch bark tar at the Mesolithic site of Star Carr.

*Ancient Biomolecules* **2**(1), 69-80 (1998).

1. Aveling, E. & Heron, C. Chewing tar in the early Holocene: an archaeological and ethnographic evaluation. *Antiquity* **73**(281), 579-84 (1999).
2. Schwartz, M. & Hollander, D. Annealing, distilling, reheating and recycling: bitumen processing in the Ancient Near East. *Paléorient* **26**(2), 83-91 (2000).
3. Grünberg, J. M. Middle Palaeolithic birch-bark pitch. *Antiquity* **76**(291), 15-16 (2002).
4. Regert, M. Investigating the history of prehistoric glues by gas chromatography–mass spectrometry. *Journal of Separation Science* **27**(3), 244-54 (2004).
5. Wadley, L., Hodgskiss, T. & Grant, M. Implications for complex cognition from the hafting of tools with compound adhesives in the Middle Stone Age, South Africa. *PNAS* **106**(24),

9590-9594 (2009).

1. Mazza, P. P. A. et al. A new Palaeolithic discovery: tar-hafted stone tools in a European Mid-Pleistocene bone-bearing bed. *Journal of Archaeological Science* **33**(9), 1310-1318 (2006).
2. Stern, B.,Clelland, S.J., Nordby, C.C. &, Urem-Kotsou, D. Bulk stable light isotopic ratios in archaeological birch bark tars. *Applied Geochemistry* **21**(10), 1668-1673 (2006).
3. Pälsi, S. Kivikautista purupihkaa. *Suomen Museo* **1931-1932**, 64 (1932).
4. van Gijn, A. L. & Boon, J. J. in *Schipluiden: a neolithic settlement on the Dutch North Sea coast c. 3500 cal bc.* Analecta Praehistorica Leidensia 37/38 (eds Louwe Kooijmans, L. P. & Jongste, P. F. B.) 261-266 (Leiden University, Leiden, 2006).
5. Alexandersen, V. in *Huseby klev. En kustboplats med bevarat organiskt material från äldsta mesolitikum till järnålder. Bohuslän, Morlanda socken, Huseby 2:4 och 3:13, RAÄ 89*

*och 485* (ed Nordqvist, B.) 117-121 (Riksantikvarieämbetet. UV Väst, Mölndal, 2005).

1. Malmros, C. in *Huseby klev. En kustboplats med bevarat organiskt material från äldsta mesolitikum till järnålder. Bohuslän, Morlanda socken, Huseby 2:4 och 3:13, RAÄ 89 och 485*

(ed Nordqvist, B.) 122-131 (Riksantikvarieämbetet. UV Väst, Mölndal, 2005).

1. Mathews, J. P. & Schultz, G. P. *Chicle. The chewing gum of the Americas, from the ancient Maya to William Wrigley* (University of Arizona Press, Tucson, 2009).
2. Wärmländer, S. K. T. S., Sholts, S. B., Erlandson, J. M., Gjerdrum, T. & Westerholm, R. Could the Health Decline of Prehistoric California Indians be Related to Exposure to Polycyclic Aromatic Hydrocarbons (PAHs) from Natural Bitumen? *Environmental Health Perspectives* **119**, 1203-2107 (2011).
3. Paraschos, S., Mitakou, S.-L. & Skaltsounis, A. Chios Gum Mastic: A Review of its Biological Activities. Current Medicinal Chemistry **19**(14), 2292-2302 (2012).
4. Nordmann, P. *Finnarne i mellersta Sverige* (Thesis, Helsingfors, 1888).
5. Demant-Hatt, E. *Med Lapperne i Højfjeldet* (Nordiska Bokhandeln, Stockholm, 1913).
6. Vilkuna, K. Muutamia tietoja purupihkan käytöstä Suomessa. *Suomen Museo* 1927, 59- 60 (1927).
7. Sirelius, U. T. *Suomen kansanomaista kulttuuria II. Suomalais-ugrilainen seura* (Helsinki, 1921).
8. Österberg, E. Koivutuohitervan valmistus kivikautisin menetelmin ja prosessin jäljet

arkeologisessa löytöaineistossa. *Muinaistutkija* **2002**(2), 38-45 (2002).

1. Osipowicz, G. A method of wood tar production, without the use of ceramics. EuroREA **2**, 11–17 (2005).
2. Pollard, A. M. & Heron, C. *Archaeological Chemistry: Edition 2* (The Royal Society of Chemistry, Cambridge, 2008).
3. Groom, P., Schenck, T. & Pedersen, G. M. Experimental explorations into the aceramic dry distillation of *Betula pubescens* (downy birch) bark tar. *Archaeological and*

*Anthropological Sciences* **7**(1), 47-58 (2013).

1. Kozowyk, P. R. B., Soressi, M., Pomstra, D. & Langejans, G. H. J. Experimental methods for the Palaeolithic dry distillation of birch bark: implications for the origin and development of Neandertal adhesive technology. *Scientific Reports* **7**, 8033 (2017).
2. Eriksson, G. Part-time farmers or hard-core sealers? Västerbjers studied by means of stable isotope analysis. *Journal of Anthropological Archaeology* **23**(2), 135-162 (2004).
3. Eriksson, G. et al. Diet and mobility among Mesolithic hunter-gatherers in Motala (Sweden) - The isotope perspective. *Journal of Archaeological Science: Reports* **17**, 904-918 (2018).
4. Eggen, I. & Nymoen, P. *Funnsted for mesolittiske skjeletter Hummervikholmen av Hallandvik, 32/69, Søgne kommune, Vest-Agder* (Museum of Cultural History, University of Oslo, Oslo, 2014).
5. Nymoen, P. *Beretning fra arkeologisk registrering under vann årene 1994, 1995, 1996, 1997 Hummervikholmen, Søgne i Vest-Agder* (Norsk Maritimt Museum, Oslo 2014).
6. Apel, J. & Storå, J. in *Arkeologi på Gotland 2: Tillbakablickar och nya forskningsrön* (eds Wallin, P. & Martinsson-Wallin, H.) 9-18 (Uppsala university, Uppsala 2017)
