# Supplementary material for: Ancient DNA from mastics solidifies connection between material culture and genetics of mesolithic hunter–gatherers in Scandinavia
Source: Commun Biol. 2019 May 15;2:185. doi: 10.1038/s42003-019-0399-1 (PMC6520363; doi:10.1038/s42003-019-0399-1)
Supplement: Supplementary file 6 — Description of Additional Supplementary Files [file 42003_2019_399_MOESM6_ESM.docx]

Description of the Additional Supplementary Data files

**Supplementary Data 1.** Sums and percentages of the central attributes associated with the morphology of blades in the Huseby Klev assemblage. By Hege Damlien.

**Supplementary Data 2.** Sums and percentages of the central blade attributes associated with knapping technique in the Huseby Klev assemblage. By Hege Damlien.

**Supplementary Data 3.** Description of the used mastics samples. By Bengt Nordqvist.
